# DEMO-EMol: modeling protein-nucleic acid complex structures from cryo-EM maps by coupling chain assembly with map segmentation

**DOI:** 10.1093/nar/gkaf416

**Published:** 2025-05-14

**Authors:** Ziying Zhang, Liang Xu, Shuai Zhang, Chunxiang Peng, Guijun Zhang, Xiaogen Zhou

**Affiliations:** College of Information Engineering, Zhejiang University of Technology, Hangzhou 310023, China; College of Information Engineering, Zhejiang University of Technology, Hangzhou 310023, China; College of Information Engineering, Zhejiang University of Technology, Hangzhou 310023, China; Department of Biological Chemistry, University of Michigan, Ann Arbor, MI 48109, United States; College of Information Engineering, Zhejiang University of Technology, Hangzhou 310023, China; College of Information Engineering, Zhejiang University of Technology, Hangzhou 310023, China

## Abstract

Atomic structure modeling is a crucial step in determining the structures of protein complexes using cryo-electron microscopy (cryo-EM). This work introduces DEMO-EMol, an improved server that integrates deep learning-based map segmentation and chain fitting to accurately assemble protein–nucleic acid (NA) complex structures from cryo-EM density maps. Starting from a density map and independently modeled chain structures, DEMO-EMol first segments protein and NA regions from the density map using deep learning. The overall complex is then assembled by fitting protein and NA chain models into their respective segmented maps, followed by domain-level fitting and optimization for protein chains. The output of DEMO-EMol includes the final assembled complex model along with overall and residue-level quality assessments. DEMO-EMol was evaluated on a comprehensive benchmark set of cryo-EM maps with resolutions ranging from 1.96 to 12.77 Å, and the results demonstrated its superior performance over the state-of-the-art methods for both protein-NA and protein–protein complex modeling. The DEMO-EMol web server is freely accessible at https://zhanggroup.org/DEMO-EMol/.

## Graphical Abstract

**Graphical Abstract:**
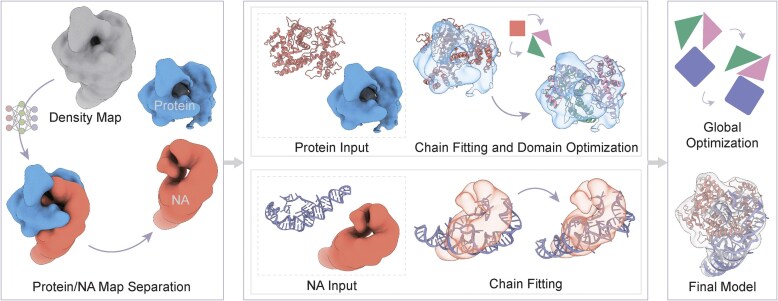

## Introduction

Accurate modeling of macromolecular structures is essential for understanding their biological functions. In recent years, cryo-electron microscopy (cryo-EM) has emerged as a powerful and increasingly popular technology for macromolecular structure determination [1–4]. A critical step in cryo-EM-based structural determination involves constructing atomic structures from cryo-EM density maps [5]. However, limited resolution of maps and interference from noise significantly complicate the high-accurate construction of atomic structures, particularly for protein–nucleic acid (NA) complexes [6].

Over the past decade, numerous methods have been developed for structural modeling from cryo-EM density maps, which can be broadly classified into two main categories: *de novo* modeling and structure-fitting modeling [7]. De novo methods usually construct models directly from density maps without relying on any prior structural knowledge. Representative methods include MAINMAST [8], DeepTracer [9], Cryo2struct [10], ModelAngelo [11], and EMRNA [12]. Although these approaches can yield highly accurate structures, their performance usually depends on the resolution and quality of the density maps [7]. In contrast, structure-fitting methods build models by fitting previously determined structures or models generated by structure prediction tools, such as AlphaFold [13, 14] and I-TASSER [15], into the density map. Representative tools include Situs [16], Phenix [17], EMBuild [18], DiffModeler [19], and DEMO-EM [20]. These approaches are applicable to both high-resolution and intermediate-resolution maps, but the performance is frequently influenced by the quality of the starting model. It is noteworthy that the majority of *de novo* and structure-fitting methods have been designed primarily for modeling proteins or NAs individually, significantly limiting their effectiveness when applied to protein–NA complexes.

DEMO-EM is a structure-fitting modeling method developed by our research team, specifically designed for multi-domain protein modeling from high-to-medium resolution (<10 Å) cryo-EM maps. Taking advantage of the hierarchical protocol that combines domain-level fitting with flexible assembly guided by map restraints and inter-domain potentials, DEMO-EM outperforms other cryo-EM-based structure modeling methods. Owing to its robust performance and user-friendly design, the DEMO-EM server has built models for more than 1000 users from 80 countries and regions. However, DEMO-EM’s modeling strategy is specifically designed for multi-domain modeling, which limits its effectiveness in modeling complexes involving protein–NA or protein–protein interactions.

In this work, we developed DEMO-EMol, an improved method for constructing high-accuracy protein–NA complex models from cryo-EM maps. DEMO-EMol integrates deep learning-based segmentation of protein and NA map regions with chain- and domain-level intertwined model-to-map fitting procedure guided by map constraints and physical potentials. Comprehensive test results on a large-scale benchmark dataset demonstrate the superior performance of DEMO-EMol in modeling both protein–NA and protein–protein complexes. DEMO-EMol is freely accessible through an online server at https://zhanggroup.org/DEMO-EMol, providing researchers with an efficient and user-friendly platform for accurate structure modeling from cryo-EM maps.

## Material and methods

### Pipeline of DEMO-EMol server

**Figure 1.**
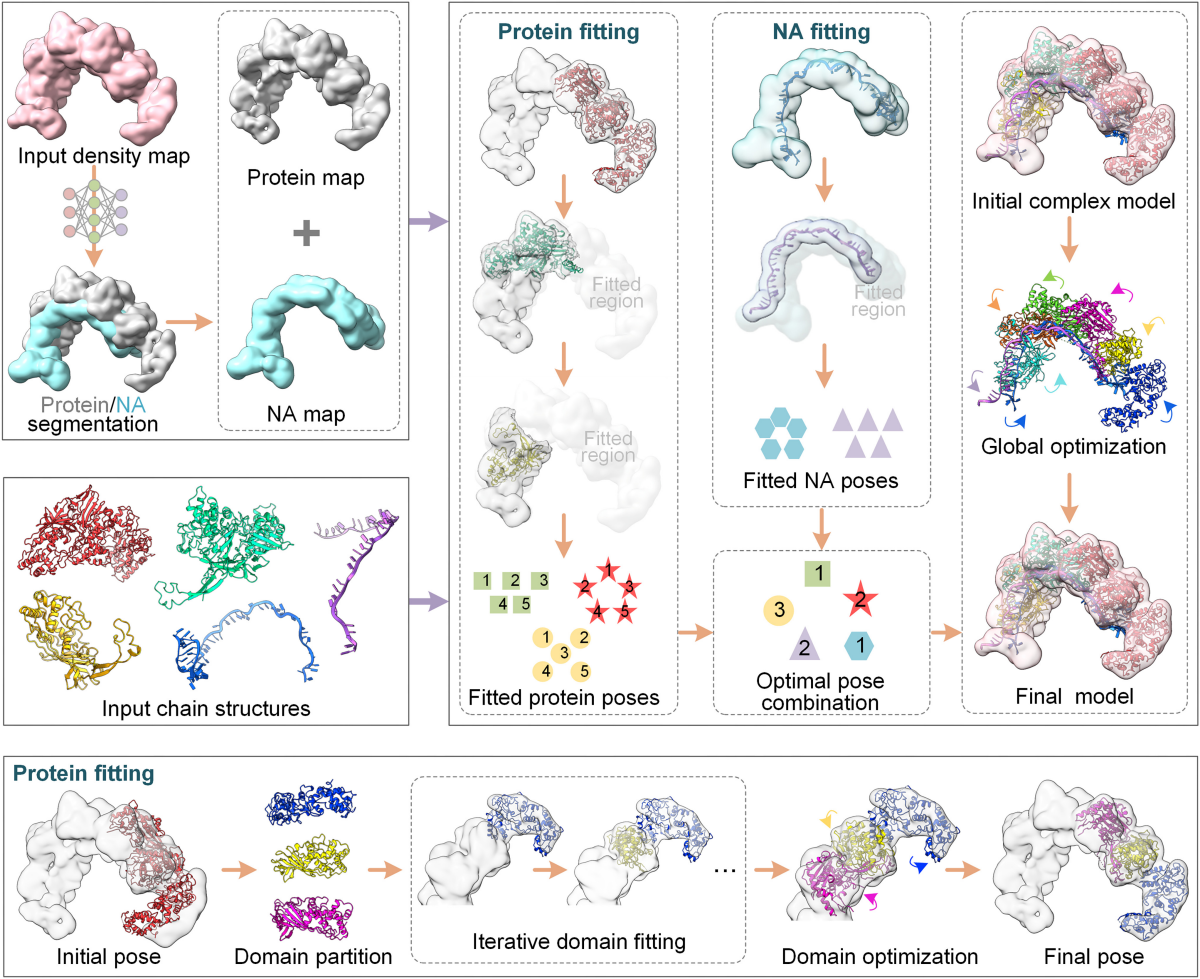
Workflow of the DEMO-EMol server.

DEMO-EMol integrates map segmentation with an iterative structure fitting and assembly process to automatically construct protein complex structures. As illustrated in Fig. 1, starting from the density map and individual chain models, the protein and NA regions are segmented from the input map using deep learning, generating a protein map and a NA map. Subsequently, protein and NA chain models are independently fitted into their corresponding segmented maps by searching their optimal poses (rotation-translation parameters) using the Limited-memory Broyden-Fletcher-Goldfarb-Shanno (L-BFGS) algorithm, where an additional domain-level optimization is applied to the protein chain fitting. To improve fitting accuracy and reduce the search space, a map masking strategy is employed to mask the map regions already matched with chain models. Once all chain models are fitted, the complex model is constructed by identifying the optimal combination of all chain poses, followed by a global optimization.

### Density map segmentation

For density maps containing both proteins and NAs, we employ the U-Net++ architecture from EMNUSS [21], which has demonstrated superior performance in semantic segmentation tasks, to accurately distinguish regions corresponding to proteins and NAs in the map. The workflow is illustrated in Supplementary Fig. 1. The training dataset is derived from the first stage dataset of EM2NA [12], comprising 156 maps for training and 39 for validation. To prepare the training data, the voxel size of the map is unified to 1 Å through trilinear interpolation, and density values below the recommended contour level are set to 0. Subsequently, standardization and normalization are performed, where 0 and the 98th percentile density value are utilized as the minimum and maximum values for normalization, respectively, and any density values above the 98th percentile density value are set to 1. Each voxel of the map is assigned a label based on the spatial proximity to the backbone atoms within 3Å of the PDB structures. Voxels closest to protein atoms are labeled as 0, those closest to NA atoms are labeled as 1, and other voxels are labeled as 2. All maps are cut into multiple overlapping chunks of size 64 × 64 × 64 Å^3^ by slicing along three axes with a step size of 32 Å. The weighted CrossEntropyLoss [22] is employed as the loss function, and the training is carried out for 200 epochs. The learning rates is automatically adjusted based on the performance on the validation set. The initial and minimum learning rate are set to 5e-4 and 1e-6, respectively. The model with the lowest validation loss is selected as the final model.

### Structure fitting and assembly

DEMO-EMol sequentially fits chain models into their corresponding segmented map in descending order of chain sequence length by searching for their optimal poses within the map through the L-BFGS algorithm. Since L-BFGS is a local optimization method, the quality of the final solution heavily depends on the initial solution. To mitigate this problem, we employ multiple initial poses for each chain fitting. The translation parameters of the initial poses are determined based on the gyration radius of both the map and the model, while the rotation parameters are selected by enumerating Euler angles [20] (Supplementary Fig. S2). Each initial pose is subjected to an independent rigid-body L-BFGS optimization. The optimization is guided by a composite scoring function that integrates both global and local model-to-map correlation coefficients (CCs) [23], as well as the Fourier Shell Correlation (FSC), to mitigate overfitting to high-resolution maps. The local CC is calculated for each residue using a nine-residue fragment centered at the residue, while the global CC is computed for the entire model (Supplementary Text S1). The top five poses with the highest correlation are selected for further processing. For protein chains, the domain-based flexible fitting strategy is applied to the rigidly fitted model, which involves independently fitting each domain into the map or refining the position and orientation of each domain (Supplementary Fig. S3). During the fitting process, we first determine chains that can be well fitted, and the map regions corresponding to these chains are masked by setting the density values to 0. Here, a chain is considered well-fitted if its correlation exceeds a predefined threshold, which is initially set at 0.6. Chains with correlations below the threshold undergo further iterative fitting by gradually reducing the threshold (Supplementary Fig. S4). After all chain models are fitted into the map, an initial complex model is constructed by identifying the optimal combination of all chain poses using a differential evolution algorithm [24], where each chain model is kept rigid. The process is guided by an energy function consists of the density correlation and the steric atom clash score [25]. Finally, the initial complex model undergoes a domain-level flexible refinement, in which the positions and orientations of all domains are simultaneously optimized to produce the final complex structure. As NA chains typically lack well-defined structural domains, they are preserved as rigid bodies during the entire assembly process.

## Web server

### Server input

The DEMO-EMol server requires three mandatory inputs including a cryo-EM density map along with its resolution and a compressed file containing PDB-format chain structures for assembly (label 1, Supplementary Fig. S5). If users only have sequences, they are advised to submit individual chain sequences to structure prediction servers such as AlphaFold3 (https://alphafoldserver.com/) or D-I-TASSER (https://zhanggroup.org/D-I-TASSER/) to obtain predicted structures. Double-stranded NAs can be provided as a single PDB file, in which case the algorithm will treat them as a rigid body to preserve base pairing during model assembly. Users are encouraged to provide an email address (label 2, Supplementary Fig. S5) to receive a notification containing a link to the results page once the job is completed. Additionally, users may specify the name of the target protein (label 3, Supplementary Fig. S5); if not provided, the protein will be automatically named “query protein” by default.

We offer two options for setting map parameters. The first option is the voxel size, which is set to 2Å by default. Users can adjust this value by entering a custom value in the box (label 4, Supplementary Fig. S5). A larger voxel size can reduce runtime but may affect modeling accuracy, while a smaller voxel size has the opposite effect. The second option is the contour level, which serves as a density threshold to filter out potential noise in the map. By default, the contour level is set to 0 (label 5, Supplementary Fig. S5), but users can modify it by inputting a different value in the box based on the recommended value from EMDB [26] or their experience.

Additionally, the server provides two advanced options (label 6, Supplementary Fig. S5) of the assembly to enable users to customize their experiments: [1] whether to perform domain-level assembly and optimization, and [2] whether to use iterative strategy. By default, both options are set to “YES” to activate the corresponding strategies. While users can select “NO” to deactivate a specific strategy, this is generally not recommended. For further clarification on these settings and options, users can click the “Explanation” or the “?” icon adjacent to each option to access detailed descriptions.

Once all inputs and settings are completed, users can submit the job by clicking the “Run DEMO-EMol” button or reset the form using the “Clear form” button (label 7, Supplementary Fig. S5). The browser will direct to a new page (Supplementary Fig. S6) containing confirmation of the job id, the submitted input file, and an estimated runtime once the job is successfully submitted. This page will automatically refresh, and all results will be displayed when the job is finished. Users are advised to bookmark this page for easy access to their results.

### Server output

**Figure 2.**
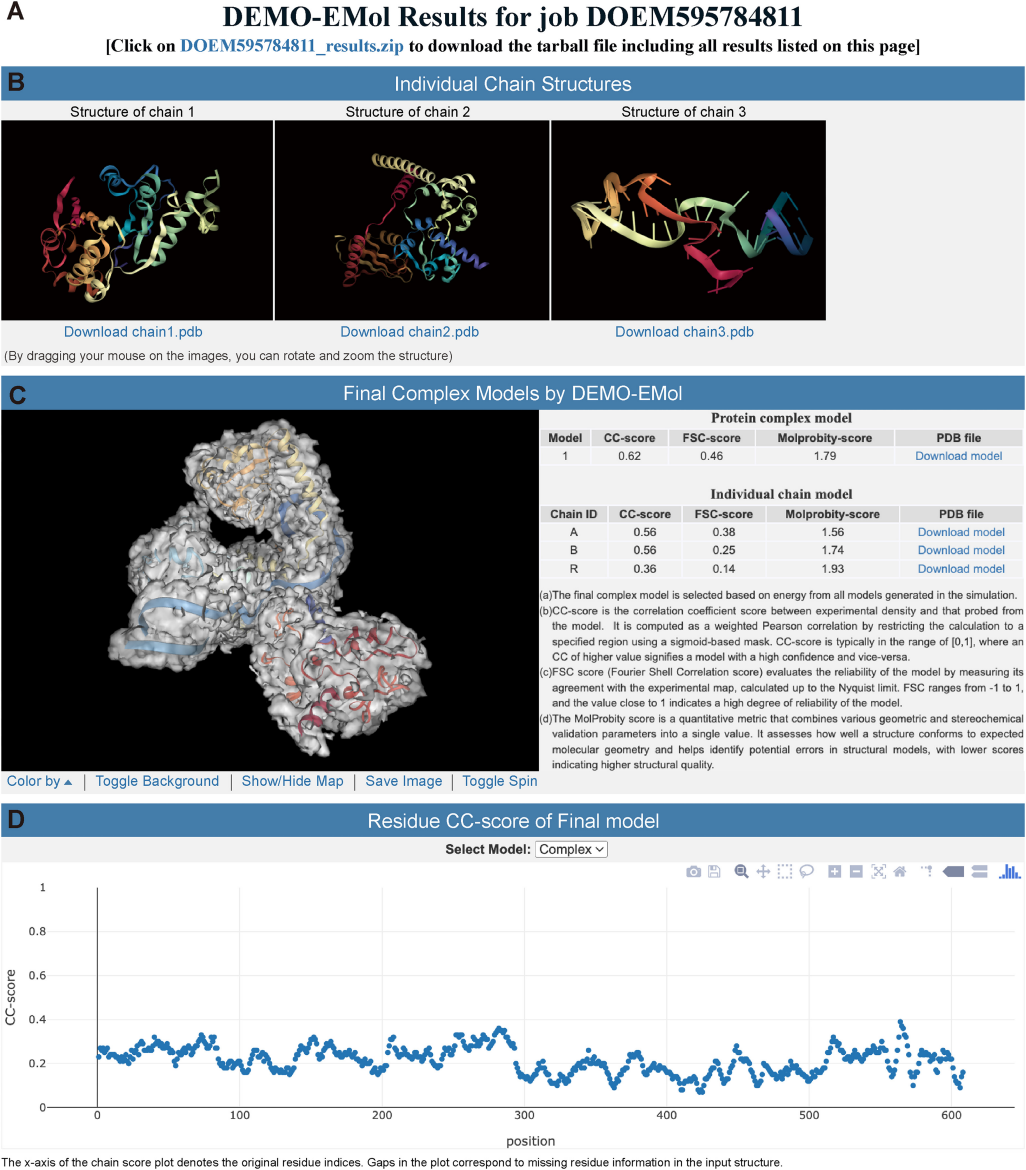
Example of the DEMO-EMol results page. (**A**) The title of the results page and the download link for all results presented on the page. (**B**) 3D visualization of users submitted chain models. (**C**) Visualization of the DEMO-EMol assembled model and the overall quality assessment. (**D**) Scatter plot of per-residue CC scores for the final model and chain models.

The DEMO-EMol server provides visualization results and model quality analyses. The results page consists of four key sections, including downloadable results file information, visualization of user submitted chain models, visualization of the DEMO-EMol assembled model along with an overall quality assessment, and a scatter plot of the per-residue CC score of the constructed model. Fig. 2 illustrates an example from *7SK core RNP with linear RNA* (EMD-25197, PDBID: 7SLP) [27], where the model is assembled by DEMO-EMol using the default settings. The result page of the example can be accessed via https://zhanggroup.org/DEMO-EMol/exampleoutput.

As shown in Fig. 2A, the first section shown at the top of the results page displays the target job ID along with a link allowing users to download a compressed file containing all results presented on the page. The results will be retained on the server for one month. Users are recommended to download the results to their local computers.

The second section provides the 3D visualization of user submitted chain models for assembly (Fig. 2B). Each chain model is displayed using an independent NGLviewer applet [28]. Users can rotate and move the model by dragging the mouse on the corresponding image, and the chain model can be downloaded by clicking the “Download chainX.pdb” link located below the image.

The third section (Fig. 2C) presents the DEMO-EMol assembled model, which is visualized in a NGLviewer applet on the left panel. Users can move and zoom the model using the mouse. Below the panel, several options are provided for users interact with the image, including “Color by” to select a coloring scheme for structural units, “Toggle Background” to switch between black and white backgrounds, “Show/Hide map” to control the visibility of the density map, “Save Image” to capture and save the current image as a PNG file, and “Toggle Spin” to enable or disable automatic model rotation. The right panel presents the model’s quality assessment, including CC-score, FSC score, and Molprobity-score [29] for both the complex model and individual chain models. Users can download the model by clicking the “Download model” link in the last column of the table. Detailed explanations of these evaluation metrics are provided below the panel. In addition to the CC-score and FSC-score reported on the server, users can further assess the quality of the assembled complex model using other model-to-map correlation metrics, such as Q-score [30] and EMRinger score [31].

The last section (Fig. 2D) presents a scatter plot illustrating the residue-level CC-score of the DEMO-EMol constructed model. Each point on the scatter plot corresponds to a specific residue. Users can hover over a point on the scatter plot of the complex model to view the residue's index and its associated CC-score. Meanwhile, the selected residue will be highlighted in spheres format in the left panel of the second section. Additionally, an interactive control panel appears in the upper right corner when the mouse is positioned over the plot. Users can click the corresponding icon to further analyse and view the scatter plot. Since residues in the complex model plot are indexed by merging all chains, a dropdown menu is provided to allow users to view each individual chain model separately with its original residue index.

## Validation

### Benchmark set

To verify the performance of DEMO-EMol, we evaluated it on a comprehensive benchmark set comprising 97 non-redundant density maps collected from EMDB (Supplementary Text S2). This dataset includes 49 protein–NA complexes (Supplementary Table S1) and 48 protein–protein complexes (Supplementary Table S2), with resolutions ranging from 2.41 to 12.77 Å and up to 18 chains in a single complex. For each query, the chain models used for assembly were independently generated using AlphaFold3, where all templates released prior to the query were excluded from the template library. This resulted in an average TM-score [32] of 0.81. Here, TM-score is a widely used metric to evaluate the topological similarity between structures, ranging from 0 to 1, with values closer to 1 indicating higher structural similarity (Supplementary Text S3).

### Protein–NA complexes assembly

**Figure 3.**
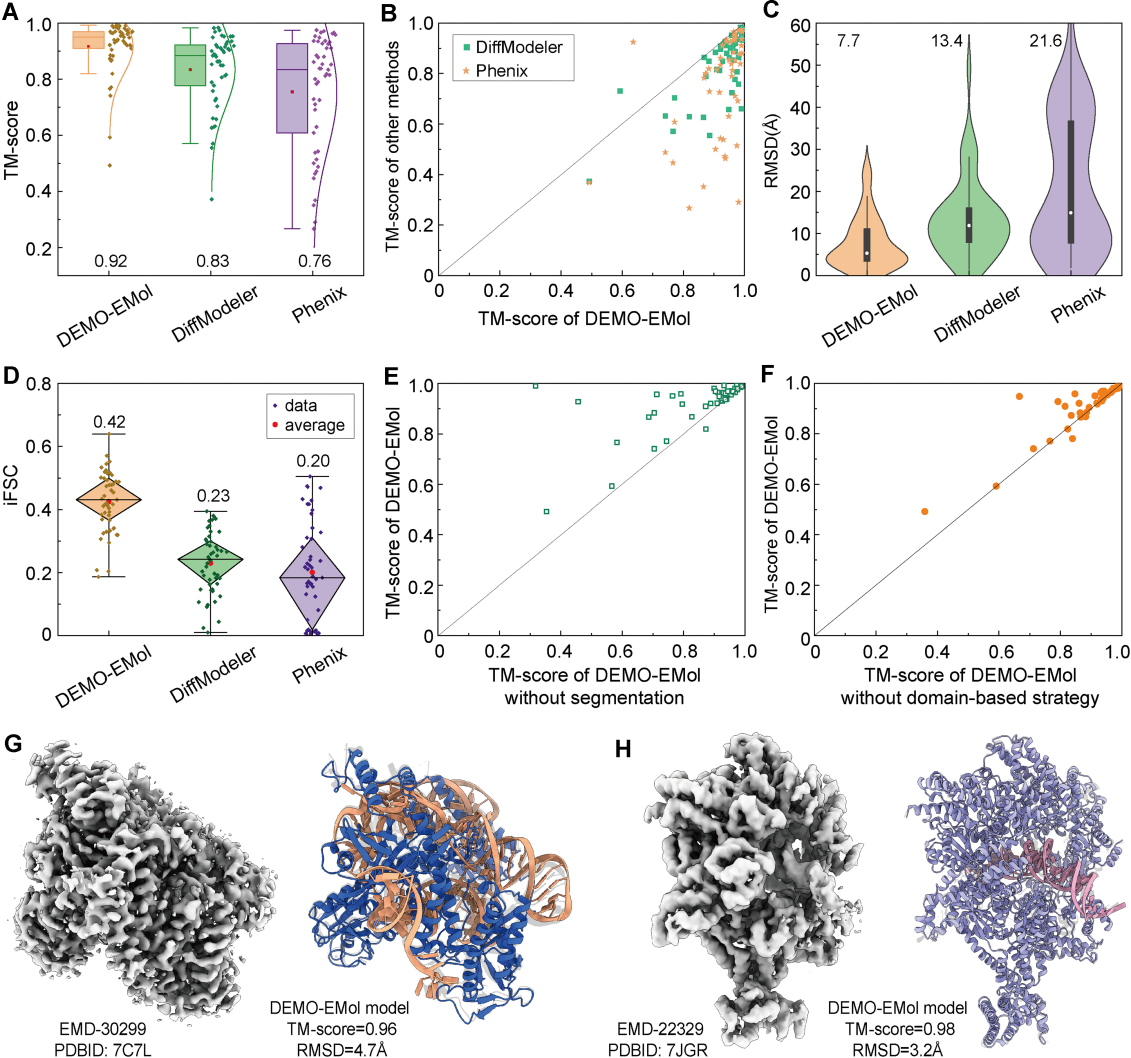
Results for 49 protein–NA complexes. (**A**) Boxplot of the TM-scores for models by different methods. The box spans the lower to upper quartiles, with the horizontal line representing the median and the red square indicating the mean (0.92, 0.83, and 0.76). The whiskers extend to the 5th and 95th percentiles. (**B**) Comparison of the TM-scores between DEMO-EMol and other methods. (**C**) Distribution of RMSDs for models constructed by different methods. Vertical lines indicate outliers (1.5), while white circles represent the means (7.7, 13.4, and 21.6). The black boxes span the 25th to 75th percentiles, and the violin plot shape illustrates the overall distribution. (**D**) Boxplot of iFSC values for models obtained using different methods. The diamond-shaped box represents the range from the lower to upper quartiles, with the horizontal line indicating the median and the red circles denoting the means (0.42, 0.23, and 0.20). Whiskers extend to the 5th and 95th percentiles. (**E** and **F**) are scatter plots of TM-scores from ablation experiments. (**G**) and (**H**) present two representative examples. The left side shows the density map, while the right side compares the DEMO-EMol constructed model (top) with the deposited structure (bottom).

We first evaluate DEMO-EMol on the protein–NA complex dataset and compare it with two state-of-the-art protein–NA complex modeling methods: DiffModeler and Phenix, where Phenix is executed using the *phenix.dock_in_map* command (Supplementary Text S4). Fig. 3A presents the TM-scores between the deposited structures and the models constructed by different methods, indicating that DEMO-EMol demonstrated a clear advantage over other methods. Specifically, the average TM-score of models by DEMO-EMol reaches 0.92, which is 10.8% and 21.1% higher than that by DiffModeler (0.83) and Phenix (0.76), respectively. The corresponding *P*-values of Student’s *t*-test are 1.10 × 10^−7^ and 2.48 × 10^−7^, suggesting that the difference is statistically significant. These results are further confirmed by the head-to-head TM-score comparison reported in Fig. 3B, where DEMO-EMol achieves a higher TM-score for the majority of cases. In terms of RMSD, as shown in Fig. 3C, DEMO-EMol exhibits a consistent performance with that evaluated by TM-score. To further assess its effectiveness in a more realistic scenario where the deposited structure was unknown, we compute the integrated FSC [33] between the model and the density map. As shown in Fig. 3D, DEMO-EMol achieves an average iFSC of 0.42, outperforming DiffModeler (0.23) and Phenix (0.20). In addition, we evaluated the model-to-map fit quality using Q-score and CC. As shown in Supplementary Fig. S7, DEMO-EMol consistently outperforms the other methods. The detailed results for each case are listed in Supplementary Tables S4–S6.

There are two main reasons for the superior performance of DEMO-EMol compared to other methods. First, the protein/NA map segmentation allows accurate fitting of protein and NA chains into their corresponding segmented maps. As shown in Fig. 3E, the use of map segmentation leads to improved TM-scores for nearly all cases, resulting in an increase in the average TM-score from 0.83 to 0.92 and a decrease in RMSD from 14.5 to 7.7 Å (Supplementary Fig. S8). Two representative examples are presented in Supplementary Fig. S9 to highlight the effectiveness of map segmentation. Second, DEMO-EMol employs a domain-based fitting strategy to flexibly refine the domain orientation and position when they are incorrect in the input chain models. As reported in Fig. 3F, the TM-scores of 53.1% cases are improved when using the domain-based fitting strategy. On average, the TM-score is improved from 0.89 to 0.92, and the RMSD is decreased from 9.0 to 7.7 Å.

Fig. 3G and H illustrate two representative cases that DEMO-EMol successfully constructed accurate protein–NA complex models. The first example, EMD-30299, is a map with a resolution of 3.3 Å derived from *the Cas12f1-sgRNA-target DNA complex* [34]. Its corresponding deposited structure (PDBID: 7C7L) consists of two protein chains and 3 NA chains. Fig. 3G presents a comparison between the model by DEMO-EMol and the deposited structure. Starting from accurate chain models by AlphaFold3 with an average TM-score of 0.88, DEMO-EMol generated model obtains a TM-score of 0.96 and an RMSD of 4.7 Å, outperforming that of DiffModeler (0.72, 8.0 Å) and Phenix (0.79, 10.4 Å) (Supplementary Fig. S10A). This superior performance is primarily due to DEMO-EMol's ability to correctly fit chain models into their respective regions via map segmentation. In contrast, DiffModeler and Phenix misidentify the map regions for NA chains, leading to incorrect placements that also affects the fitting of protein chains. The second example, EMD-22329, is a 3.9 Å resolution map of the *Drosophila ORC bound to DNA (84 bp) and Cdc6* [35]. The corresponding deposited structure (PDBID: 7JGR) contains seven protein chains and two NA chains. The initial chain models for assembly include two inaccurate protein chain models, resulting an average TM-score of 0.67. Despite this, DEMO-EMol constructs a model closely aligned with the deposited structure (Fig. 3H), achieving a TM-score of 0.98 and an RMSD of 3.2 Å, which is also superior to models by DiffModeler (0.86, 13.7 Å), Phenix (0.29,  45.9 Å) (Supplementary Fig. S10B). The high accuracy of DEMO-EMol model is attributed not only to map segmentation but also to its domain-based fitting strategy, as evidenced by the improvement in TM-score from 0.63 to 0.94 for the protein chain in this case.

### Protein–protein complexes assembly

**Figure 4.**
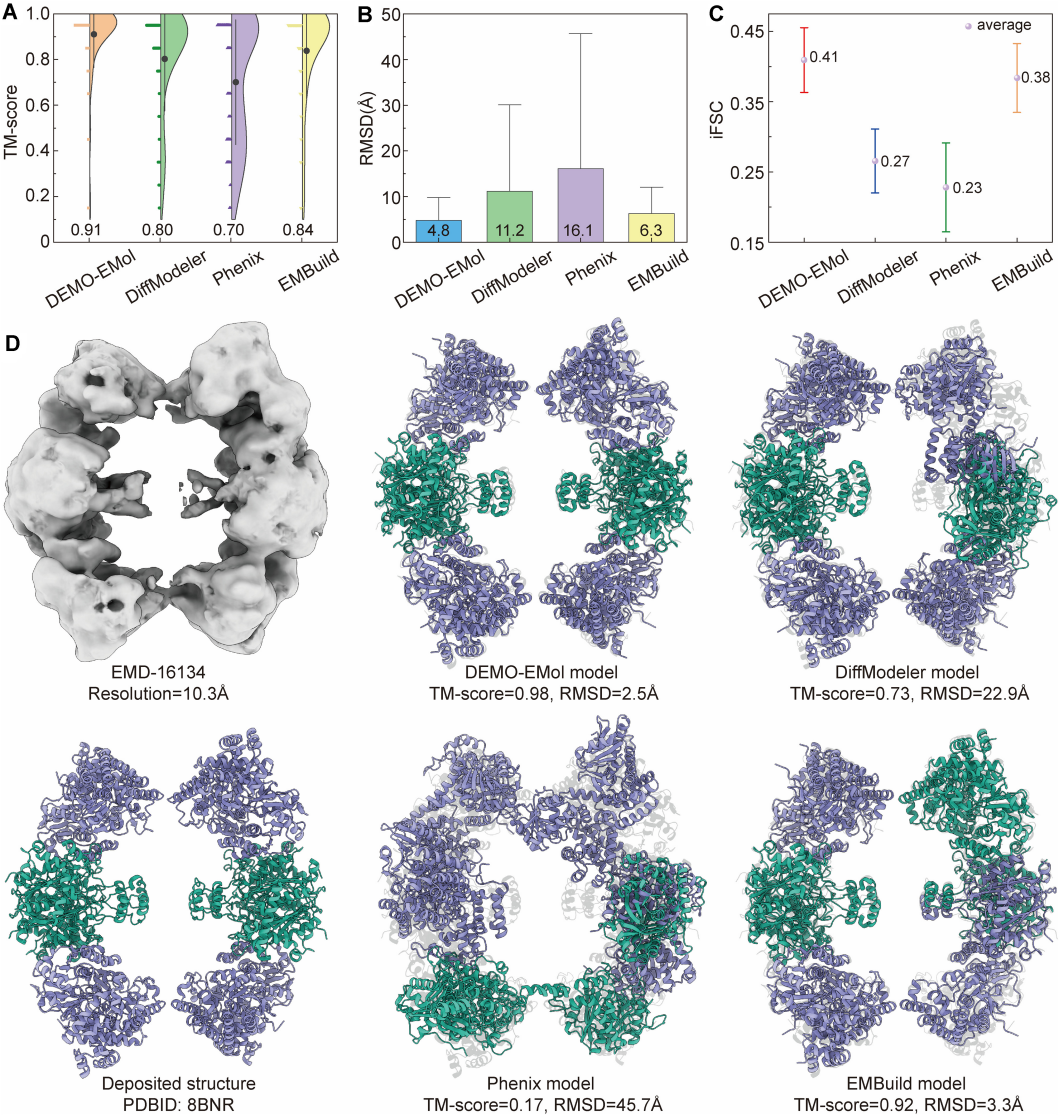
Results for 48 protein–protein complexes. (**A**) The distribution of TM-scores for models constructed by different methods. The vertical lines represent the 10th to 90th percentiles; the shape of the half-violin plot illustrates the distribution; and the black circle represents the mean. The left half of the violin plot, with various shapes, illustrates the TM-score corresponding distribution for each method. (**B**) RMSD of models constructed by different methods. Error bars indicate ± 1.0 standard deviations. (**C**) Comparison of iFSC across different methods. Whiskers represent the mean ± 95% CI, with a central sphere indicating the mean. (**D**) Comparison of models (consists of eight chains, with two types of homologous chains) by different methods with the deposited structure (bottom).

DEMO-EMol is further evaluated on the dataset of 48 protein–protein complexes. In this experiment, we also compare it with EMBuild in addition to the previously tested methods. As shown in Fig. 4A, DEMO-EMol consistently outperforms other methods. Specifically, models by DEMO-EMol achieve an average TM-score of 0.91, which is 13.8%, 30.0%, and 8.3% higher than those by DiffModeler (0.80), Phenix (0.70), and EMBuild (0.84), respectively. The corresponding *P*-values of Student's *t*-test are 1.94 × 10^−4^, 7.96 × 10^−7^, and 7.82 × 10^−3^, indicating statistically significant differences. A head-to-head TM-score comparison presented in Supplementary Fig. S11 further demonstrates that DEMO-EMol generates better models than the other methods for most test cases. As shown in Fig. 4B, DEMO-EMol achieves an average RMSD of 4.8 Å, outperforming DiffModeler (11.2 Å), Phenix (16.1 Å), and EMBuild (6.3 Å). The corresponding *P*-values from Student’s *t*-test of 5.61 × 10^−4^, 4.18 × 10^−5^, and 2.48 × 10^−1^, indicating that the differences between DEMO-EMol and DiffModeler or Phenix are statistically significant, whereas the difference compared to EMBuild is not. In terms of iFSC (Fig. 4C), CC (Supplementary Fig. S12A), Q-score (Supplementary Fig. S12B), DEMO-EMol exhibits consistent performance with that evaluated by TM-score. The detailed results for each case are reported in Supplementary Tables S7–S10. In addition, Supplementary Tables S11–S12 and Supplementary Fig. S13 specifically present the results on 20 density maps with resolutions better than 5 Å (Supplementary Table S3). The results show that DEMO-EMol outperforms DiffModeler, Phenix, and EMBuild, with corresponding Student’s t-test *P*-values of 2.57 × 10^-2^, 1.39 × 10^-3^, and 1.68 × 10^-2^ in terms of TM-score, indicating that the differences are statistically significant.

Fig. 4D presents a representative example of the accurate protein-protein complex model constructed by DEMO-EMol. This case, EMD-16134, corresponds to a 10.3 Å resolution map of *the Escherichia coli anaerobic fatty acid beta-oxidation trifunctional enzyme (anEcTFE) octameric complex* (PDBID: 8BNR, containing eight chains) [36]. Despite the low resolution, DEMO-EMol successfully builds a model with a TM-score of 0.98 and an RMSD of 2.5 Å, outperforming DiffModeler (0.73, 22.9 Å), Phenix (0.17, 45.7 Å), and EMBuild (0.92, 3.3 Å), demonstrating the robustness of DEMO-EMol in handling challenging density maps. Supplementary Fig. S14 presents two additional representative cases, further demonstrating that DEMO-EMol constructs more accurate models than the other three methods for high-resolution maps. Furthermore, Supplementary Fig. S15 shows that DEMO-EMol can successfully model a protein complex consisting of up to 24 chains. These results highlight that although DEMO-EMol is primarily designed for protein–NA complex structures assembly from cryo-EM maps, it is also capable of constructing accurate models for protein–protein complexes.

To investigate the performance of DEMO-EMol across different resolutions, we analyzed the TM-scores of DEMO-EMol models in relation to both the map resolution and the average TM-scores of the initial chain structures across the 97 cases. As shown in Supplementary Fig. S16, the TM-scores of the constructed models consistently remain above 0.8 even as the map resolution decreases. However, it is important to note that the quality of the final models is also affected by the accuracy of the initial chain structures.

## Conclusion

In this work, we present DEMO-EMol, a web server designed to construct atomic structures of protein complexes from cryo-EM density maps. As an advanced version of DEMO-EM, DEMO-EMol assembles independently modeled chain structures into accurate protein complex models by integrating deep learning-based protein/NA map segmentation with domain-based chain fitting and optimization. The DEMO-EMol server is freely accessible to the public and features an intuitive, user-friendly interface, requiring no personal information. The result page provides a visualization of the final complex model, quality assessments at both the overall and residue levels, and downloadable files containing all results. DEMO-EMol was carefully tested on a benchmark dataset comprising both protein–NA and protein–protein complexes. The results demonstrate that DEMO-EMol consistently outperforms the state-of-the-art methods in model accuracy.

While DEMO-EMol has demonstrated promising results, several aspects remain to be improved. First, DEMO-EMol currently does not support direct modeling of heterogeneous maps. Integrating domain- and secondary structure-level flexible assembly with latent information extracted from particle images via deep learning may facilitate heterogeneous structure modeling. Second, the current fitting process guided by the traditional CC is usually inefficient for cases involving a large number of chains. Developing an end-to-end model-to-map fitting approach based on local and global geometric feature matching using deep learning may significantly improve fitting efficiency. Third, the output NA models may fail to preserve base pairing in double-stranded NAs if such pairings are not provided as unified input units. Introducing explicit energy terms to enforce base pairing constraints could further improve the accuracy and plausibility of NA models. We are continuously enhancing the DEMO-EMol server and hope that it will become an essential tool for cryo-EM-based protein structure modeling and further functional research.

## Supplementary Material

gkaf416_Supplemental_File

## Data Availability

The DEMO-EMol web server is available at: https://zhanggroup.org/DEMO-EMol/. This website is free and open to all users and there is no login requirement.
