# Supplementary material for: DEMO-EMol: modeling protein-nucleic acid complex structures from cryo-EM maps by coupling chain assembly with map segmentation
Source: Nucleic Acids Res. 2025 May 14;53(W1):W228–37. doi: 10.1093/nar/gkaf416 (PMC12230720; doi:10.1093/nar/gkaf416)
Supplement: gkaf416_Supplemental_File [file gkaf416_supplemental_file.pdf]

Supplementary Information for

DEMO-EMol: Modeling protein-nucleic acid complex structures from cryo-EM maps by coupling chain assembly with map segmentation

Ziying Zhang[1,†], Liang Xu[1,†], Shuai Zhang[1], Chunxiang Peng[2], Guijun Zhang[1,*] and Xiaogen Zhou[1,†,*]

[1]College of Information Engineering, Zhejiang University of Technology, Hangzhou 310023, China

[2] Department of Biological Chemistry, University of Michigan, Ann Arbor, MI, 48109 USA

*To whom correspondence should be addressed. Tel: +86 15257118077; Email: zxg@zjut.edu.cn.

Correspondence may also be addressed to Guijun Zhang (Email: zgj@zjut.edu.cn)

†These authors contributed equally.

Supplementary Text

Supplementary Text S1. Integrated CC-FSC Scoring Function.

To enhance fitting accuracy and reduce overfitting to high-resolution maps, we combined CC, which is more sensitive to local fitting, with FSC, which better captures global fitting quality. For the calculation of CC score, we apply a sigmoid-based soft mask to emphasize the local region surrounding the model, following the method described in the reference (Journal of Molecular Biology, 392(1):181-190, 2009)(1). We calculate both the local CC of residue fragments and the global CC of the entire model. For FSC, we compute the correlation between the Fourier transform of the model and the experimental density map using the Nyquist limit, following the reference (Nature Methods, 12(4):361-365, 2015)(2). The integrated scoring function is defined as:

$$Score = w_1 \frac{\sum_{r=1}^{L} CC_r}{L} + w_2 CC_{global} + w_3 FSC,$$

where r represents the residue index, L denotes the total number of residues in the model, CC_r refers to the local CC score calculated for the 9-residue fragment centred at residue r, and CC_{global} refers to the overall correlation between the entire model and the density map. The CC score is calculated as:

$$CC = \frac{\sum_{i=1}^{N}(\rho_E(v_i) - \overline{\rho_E})(\rho_M(v_i) - \overline{\rho_M})}{\sqrt{\sum_{i=1}^{N}(\rho_E(v_i) - \overline{\rho_E})^2 \sum_{i=1}^{N}(\rho_M(v_i) - \overline{\rho_M})^2}},$$

where N is the number of voxels with density values above a specified threshold, $\rho_E(v_i)$ is the density value of the i-th voxel in the density map, $\overline{\rho_E}$ represents the average density value of the density map, $\rho_M(v_i)$ is the density value of the i-th voxel in the density map generated according to the model, and $\overline{\rho_M}$ is the average density value of the model-derived map. $\rho_M(v_i)$ is calculated as follow:

$$\rho_M(v_i) = \sum_{j=1}^{L} m \sqrt[3]{\left(\frac{\pi}{(2.4 + 0.8R)^2}\right)^2} exp\left(-\left(\frac{\pi}{(2.4 + 0.8R)}^2\right)|v_i - x_j|^2\right),$$

where x_j is the coordinate of Cα atom (for proteins) or C4' atom (for NAs) in the j-th residue of the model, m represents the mass of the atom, R represents the resolution of the density map, and v_i represents the position of the i-th voxel. The optimal weight factors w_1, w_2, and w_3 in Score were determined by maximizing the correlation between the integrated score and Root Mean Square Deviation (RMSD) of the decoy models to the native structures based on a training set of 254 proteins(3) that are non-redundant with the benchmark set. The optimal weight values are $w_1 = 0.91$, $w_2 = 1.12$, $w_3 = 0.55$.

Supplementary Text S2. Benchmark dataset construction.

To construct the protein-nucleic acid (NA) complexes dataset, we first downloaded all density maps from EMDB, along with their corresponding deposited structures and FASTA sequences from the PDB. We then selected entries with resolutions between 2Å and 15Å that contained both protein and NA components. To remove redundancy, we implemented a two-step clustering strategy. First, Foldseek(4) was employed for initial clustering based on single-chain structural similarity (threshold = 0.5), followed by MMseqs (5) for secondary clustering using a sequence identity threshold of 0.8. From each resulting cluster, 1-2 representative structures were randomly selected to construct an initial dataset. We further refined the dataset by excluding maps with individual chain lengths below 20 residues and those with a correlation coefficient to the deposited structure of less than 0.7. This process yielded a high-quality test set of 61 protein-NA complexes. Subsequently, the individual chains were modeled using AlphaFold3. After excluding 12 cases where the comparison methods failed to produce final models, a final set of 49 protein-NA complexes was retained. The final non-redundant protein-NA complex dataset includes density maps with resolutions ranging from 2.54Å to 5.7Å.

For the protein-protein complexes dataset, we selected density maps with resolutions between 2Å and 15Å, along with their corresponding deposited structures and FASTA sequences from the PDB, including only complexes composed exclusively of proteins. However, instead of using Foldseek for initial clustering, we directly applied MMseqs to cluster the cases based on single-chain sequence similarity, using a threshold of 0.8. All subsequent procedures were carried out in the same manner as those used for the protein-NA complex dataset. This process resulted in a high-quality test dataset comprising 62 protein complexes. For each complex, all individual chains were independently modeled using AlphaFold3. After removing 14 cases where comparison methods failed to generate final models, a total of 48 protein-protein complexes remained, with corresponding density map resolutions ranging from 2.41Å to 12.77Å.

Supplementary Text S3. TM-score.

TM-score(6) is a widely used metric to evaluate the topological similarity between structures, which can be calculated by

$$\text{TM-score}= max\left[\frac{1}{L_{target}}\sum_{i=1}^{L_{aligned}}\frac{1}{1+\left(\frac{d_i}{d_0(L_{target})}\right)^2}\right]$$

where L_{target} is the residue-level sequence length of the target protein complex, and $L_{aligned}$ is the length of the aligned residues to the deposited structure, which may differ from L_{target}, e.g., in the case threading alignment with gaps or insertions. $d_0\left(L_{target}\right) = 1.24\sqrt[3]{L_{target-15}} - 1.8$ is scale to normalize the match difference, and "max" refers to the optimized value selected from various rotation and translation matrices for structure superposition. The value of TM-score ranges in [0,1], where 1 indicates that the two structures are identical. Stringent statistics showed that TM-score>0.5 corresponds to a similarity with two structures having the same fold defined in SCOP/CATH(7).

Supplementary Text S4. Implementation of phenix.dock_in_map program

The program phenix.dock_in_map automatically docks one or more models into the map. phenix.dock_in_map works by using SSM and convolution-based shape search to find parts of the map that are similar to the model. Here's an example of how it works.

The emd_0290.map file and its corresponding resolution and the tertiary structure corresponding to each chain generated by AlphaFold3 (6hwi_chain1.pdb, 6hwi_chain2.pdb, 6hwi_chain3.pdb) were used as input files:

1. Activating the phenix configuration environment:

```
Source   /phenix/phenix_env.sh
```

2. Fitting all of chain into the density map:

```
phenix.dock_in_map   6hwi_chain1.pdb   6hwi_chain2.pdb   6hwi_chain3.pdb   emd_0290.map
resolution=7.2   nproc=4   pdb_out=phenix_new.pdb
```

Supplementary Figures

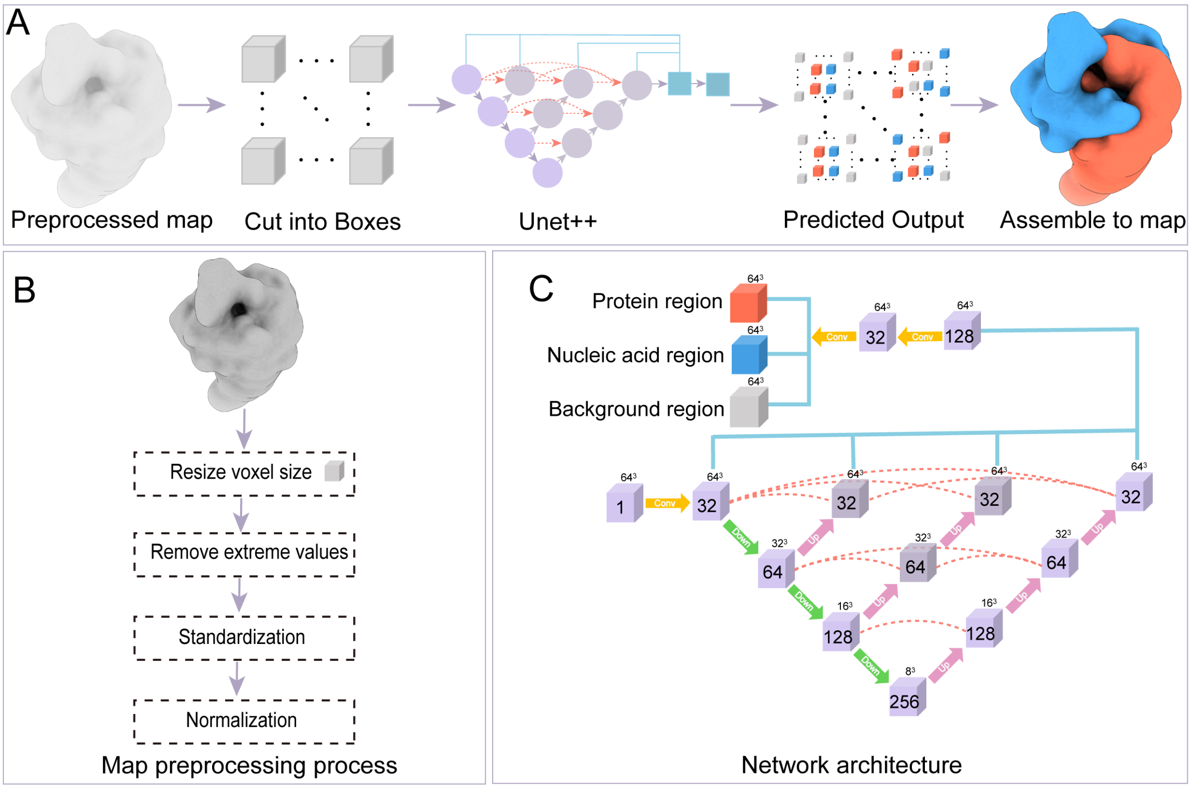

Supplementary Figure S1. Flowchart of density map segmentation Flowchart. **(A)** The pre-processed density map is divided into cubic blocks of size 64×64×64. Each block is individually processed by the network to predict the category of each voxel. Subsequently, all blocks are stitched together to obtain the predicted nucleic acid or protein regions in the density map. **(B)** The initial density map undergoes four preprocessing steps before being divided into cubic blocks for the network. First, the voxel size is adjusted to a uniform 1 Å using trilinear interpolation. Second, extreme density values are removed to eliminate outliers. Third, the density map is standardized to achieve a mean of 0 and a standard deviation of 1, which enhances network convergence. Finally, the data is normalized to ensure consistency across all density maps and to maintain uniformity throughout the network training process. **(C)** The network architecture consists of multiple interconnected layers, represented as cubes in the diagram and connected through convolution blocks. Each cube is labelled with the number of channels, while the size is indicated at the top. Yellow arrows represent convolution operations, green arrows denote downsampling, and pink arrows indicate upsampling. Pink dashed lines represent skip connections. The outputs from each layer of the U-Net are connected and concatenated via cyan-blue lines, followed by a convolution layer for final classification.

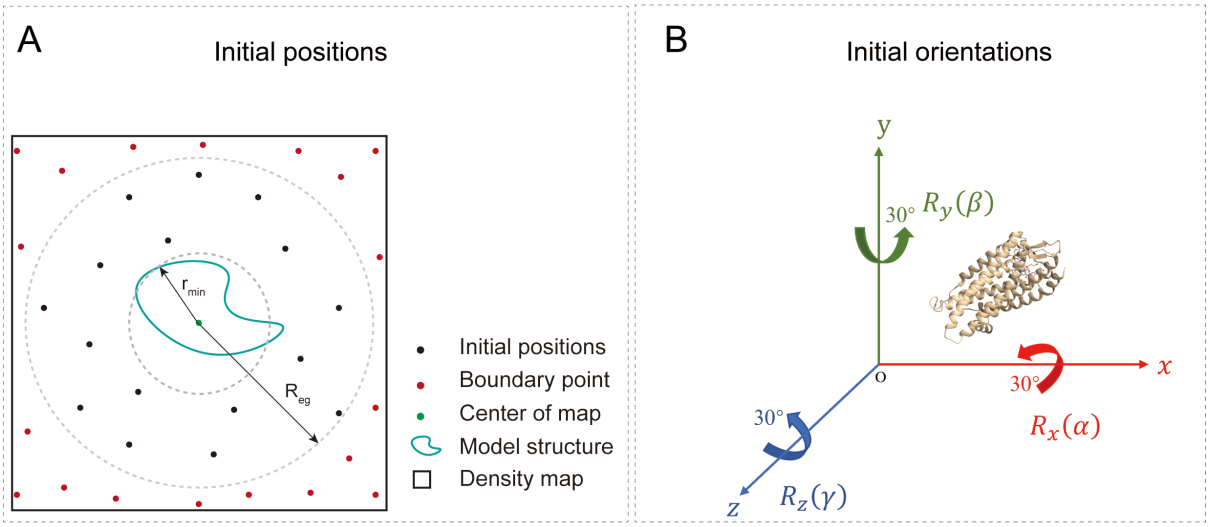

Supplementary Figure S2. Initial poses (rotation-translation parameters) generation. **(A)**The enumeration of initial positions (translation parameters) for a chain model requires that the distance between any two adjacent positions be greater than r_{min}=max(0.85r_m, r_0), where r_m represents the model's radius of gyration, r_0=5Å is the minimum allowable distance between two initial positions. Additionally, to eliminate edge positions, the maximum distance between each initial position and the center point of the density map must not exceed R_{eg}=min(max(1.1(r_v-r_m),r_0),r_v), where r_v is the radius of gyration of the density map. **(B)** The initial orientation (rotation angle) for each initial position is determined by enumerating combinations of Euler angles (α, β, γ) with a step size of 30° , Subsequently, the orientation with the higher correlation coefficient (8) and are selected as the initial orientation for each position to reduce computational costs.

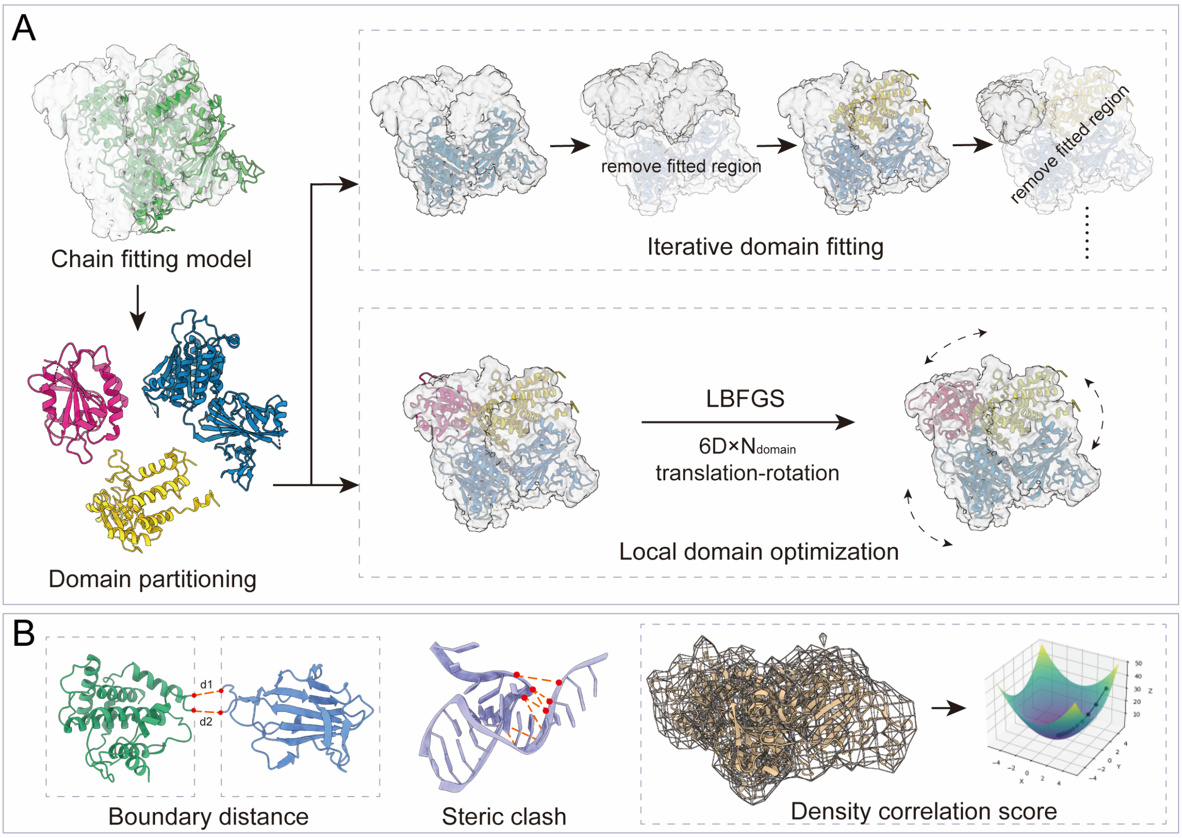

Supplementary Figure S3 Domain-based fitting strategies. **(A)** The chain is segmented into multiple domains. Proteins without homologous chains undergo iterative domain fitting. Each domain is individually matched to the density map in a long-to-short order based on sequence length, using the limited-memory Broyden-Fletcher-Goldfarb-Shanno (L-BFGS) algorithm, guided by the density correlation energy functions shown in Figure B. For proteins with homologous chains, local domain optimization is performed using the L-BFGS algorithm to refine the position and orientation of each domain within the chain, guided by the three energy functions shown in Figure B. **(B)** Three types of energy functions: Boundary distance is the connectivity between domain boundary, preventing disconnection between domain segments. The domain boundary connectivity score is calculated as $f_{bc}(m,n) = (d_{mn} - d_0)^2$, where d_0=3.8Å represents standard distance between adjacent Cα atoms. d_{mn} represents the distance between the Cα atom of the C-terminal residue of the *m*-th domain and the N-terminal residue of the *n*-th domain. The second energy function, steric clash serves as a spatial constraint between domains to prevent steric overlap. It is defined as $f_{sc}(m,n) = \sum_{i=1}^{L_m} \sum_{j=1}^{L_n} \left(min\left(0, d_{ij}^{mn} - d_{cut}\right)\right)^2$, where d_{ij}^{mn} represents the distance between the *i*-th Cα atom in the *m*-th domain and the *j*-th *C*α atom in the *n*-th domain in the structure. *Lm* and *Ln* respectively represent the number of residues in the *m*th domain and *n*th domain d_{cut} represents the minimum allowable distance between two *C*α atoms. The third energy function, Density Correlation Score (DCS), evaluates the agreement between the structure and the density map in real space, ensuring optimal structural fitting. For further details, refer to DEMO-EM(8).

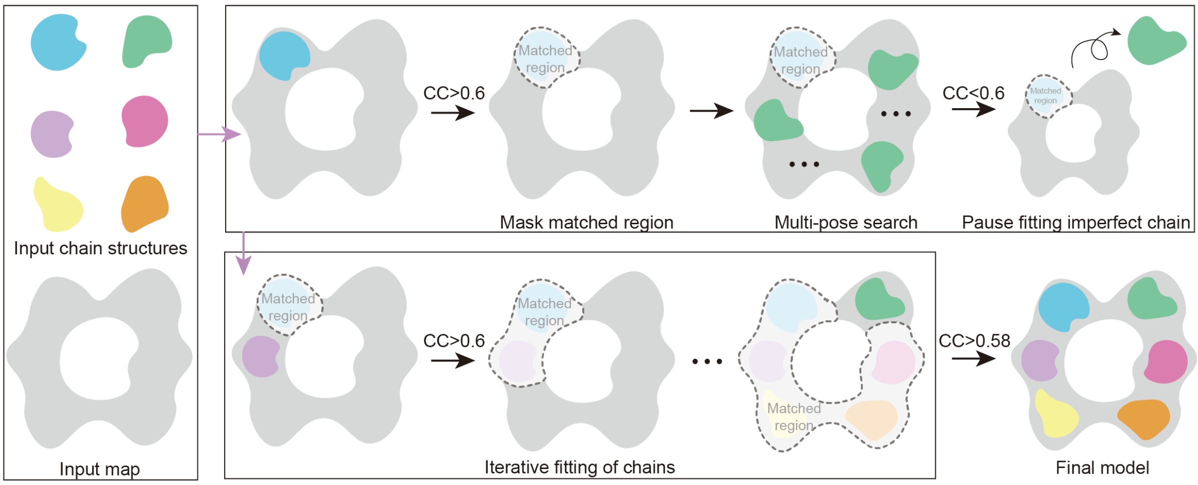

Supplementary Figure S4. Iterative fitting process combined with a masking strategy. During the fitting process, chains that are well-fitted (determined by a correlation coefficient (CC) above an initial threshold of 0.6) are retained, and their corresponding density regions are masked by setting the map values to 0. To avoid conflicts between well-fitted chains, we ensure that the fitted regions of different chains do not overlap in the density map. If chains occupy overlapping regions, only the one with the higher CC score is retained for that fitting iteration. This prevents overlapping placements and facilitates more accurate positioning of the remaining chains. Chains that initially fail to meet the correlation threshold are subjected to iterative re-fitting, in which the threshold is gradually decreased in steps of 0.02, and the process is repeated until all chains have been successfully fitted into the density map.

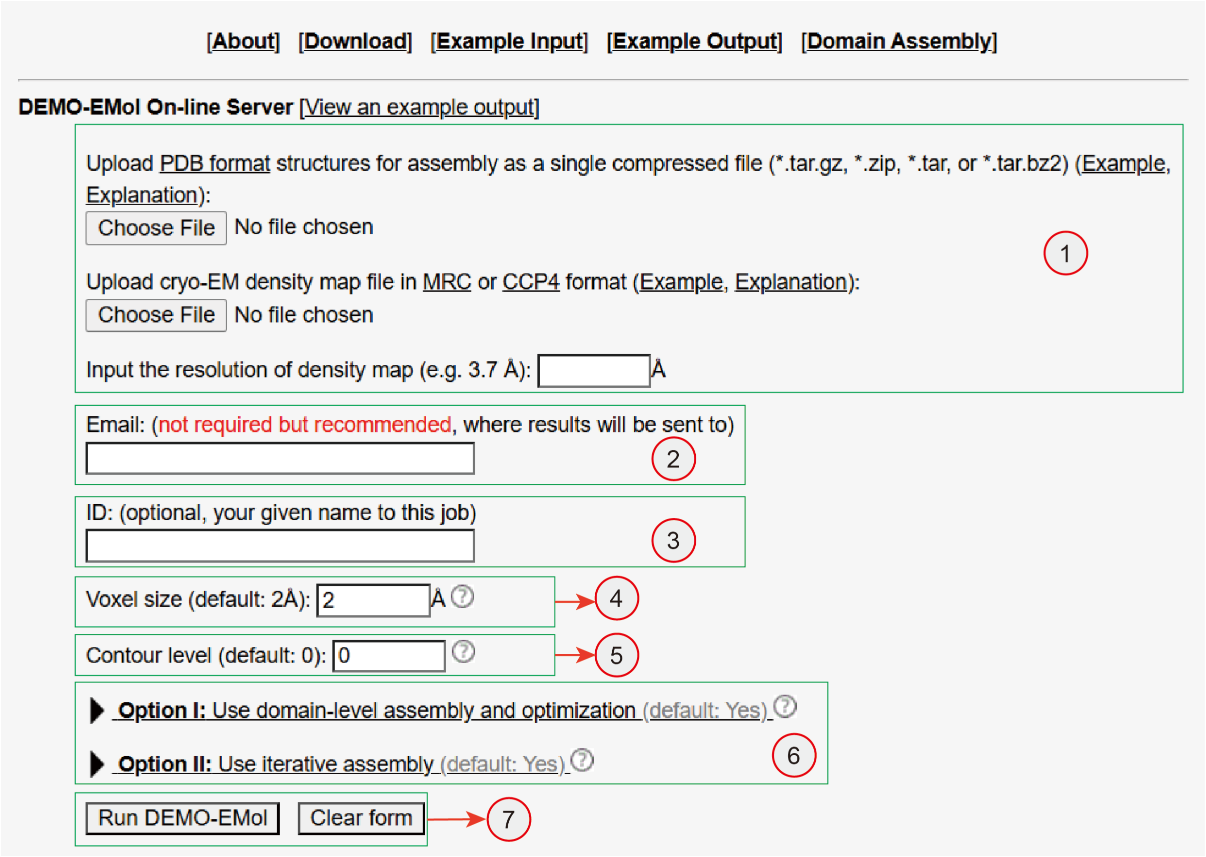

Supplementary Figure S5. Main input page of the DEMO-EMol server. **(1)** Input cryo-EM density map along with its resolution and the PDB structures for assembly. **(2)** Email address. **(3)** The name of the job. **(4)** Voxel size of the density map. **(5)** Contour level of the density map. **(6)** Two advanced options. **(7)** Submit and Reset buttons.

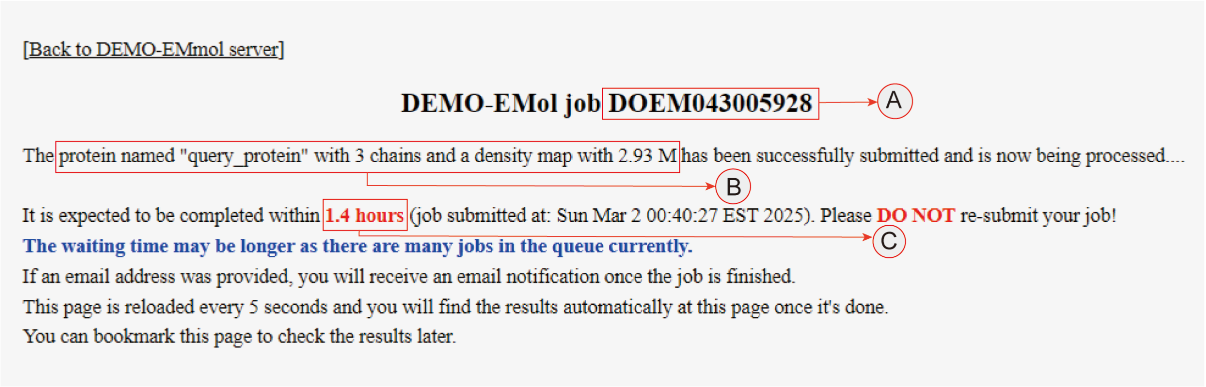

Supplementary Figure S6. Server waiting page. **(A)** Each job has a unique ID. The waiting and results page URL is https://zhanggroup.org/DEMO-EMol/output/(job_id), where (job_id) represents the unique identifier assigned to each job. **(B)** User-input information, including the target name, the number of chains to be assembled, and the density map size. **(C)** Estimated waiting time, which depends on the density map size and the number of chains to be assembled.

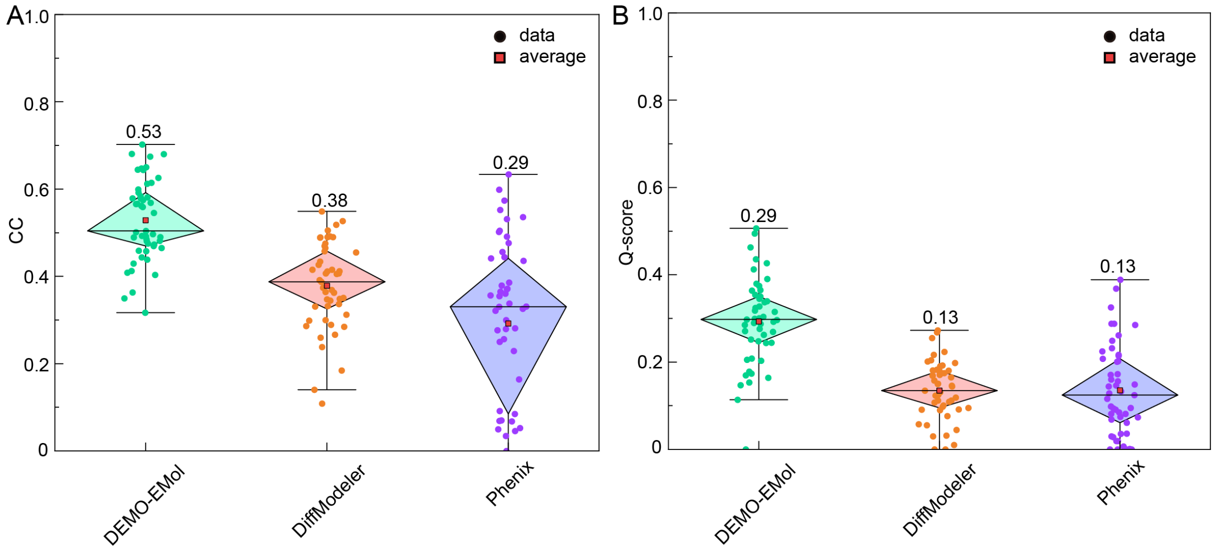

Supplementary Figure S7. Box-and-whisker plots comparing model-to-map fit metrics (CC and Q-score) across 49 protein-nucleic acid complexes. The diamond-shaped box represents the range from the lower to upper quartiles, with the horizontal line indicating the median and the red square denoting the means. Whiskers extend to the 5th and 95th percentiles. **(A)** CC. **(B)** Q-score.

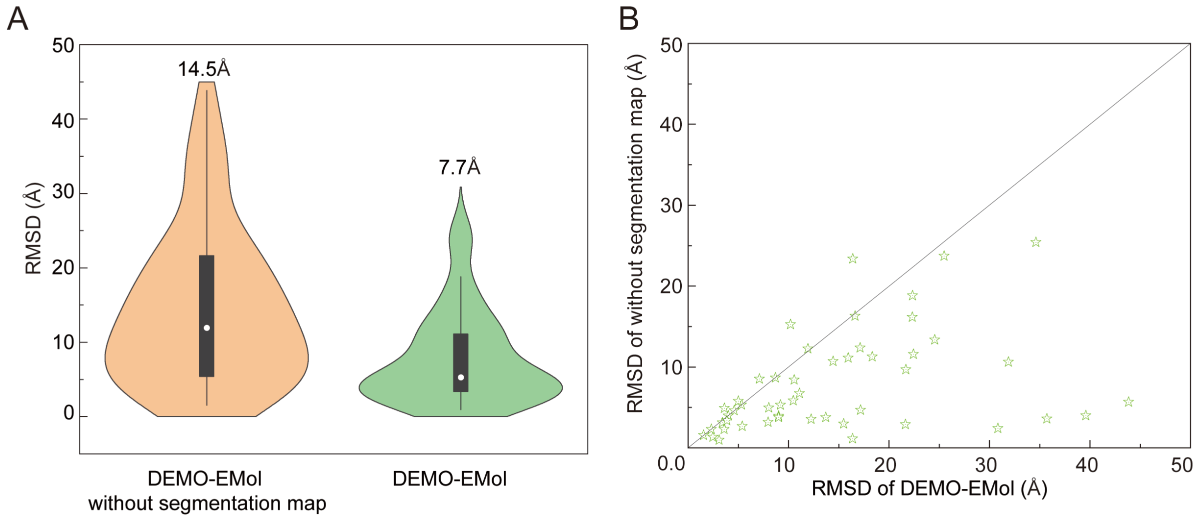

Supplementary Figure S8. Results of the map segmentation module ablation experiment on 49 maps. **(A)** Distribution of RMSDs for models constructed by DEMO-EMol with and without map segmentation. Vertical lines indicate outliers (1.5), white squares denote means, black boxes represent the 25-75th percentiles, and the violin plot illustrates the distribution. The average RMSDs are 14.5 Å (without segmentation) and 7.7 Å (with segmentation). **(B)** Comparison of RMSDs for each case between DEMO-EMol with and without map segmentation.

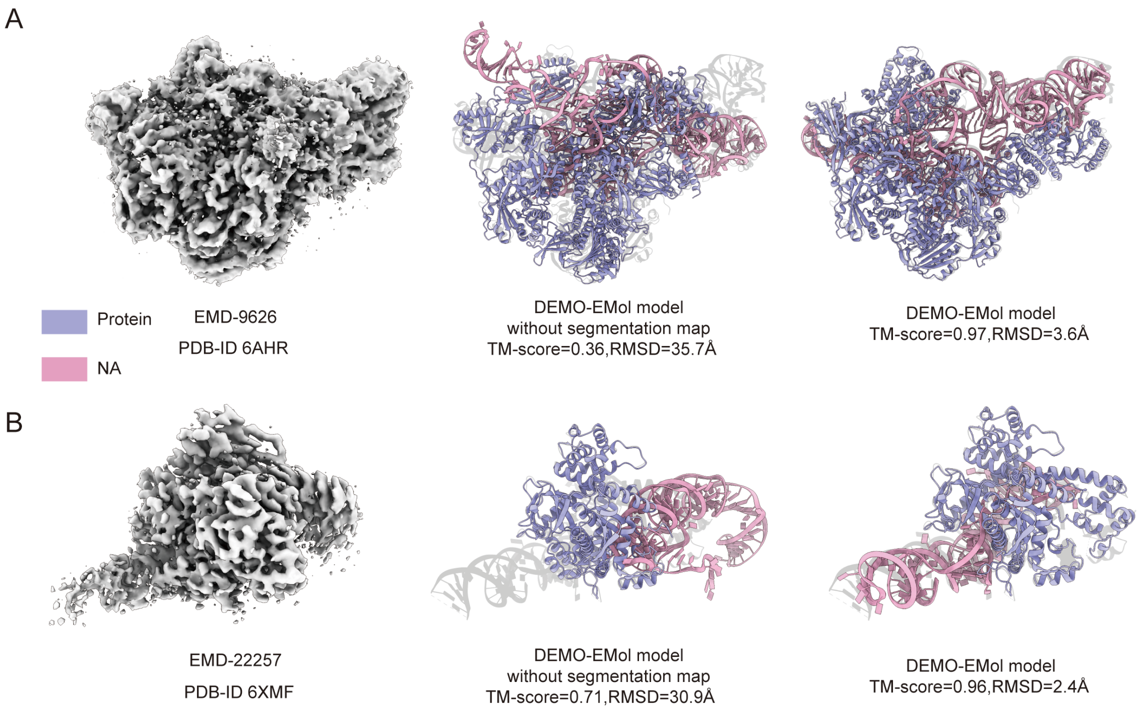

Supplementary Figure S9. Two representative cases for map segmentation. **(A)**EMD-30299, a 3.92Å resolution map derived from *human Ribonuclease P structure*(9). Its corresponding deposited structure (gray) consists of 11protein chains and 1 nucleic acid chain. After density map segmentation, the model constructed by DEMO-EMol (proteins in purple, nucleic acid chain in pink) obtained significant improvements, with the TM-score increasing from 0.36 to 0.97 and the RMSD decreasing from 35.7Å to 3.6Å. **(B)** EMD-22257: A 3.1Å resolution map corresponding to the Cryo-EM structure of the *Cas12g binary complex*(10), which includes 1 protein chain and 1 nucleic acid chain in the deposited structure (gray). The application of map segmentation led to a substantial enhancement in model quality, as evidenced by notable improvements in both TM-score (0.71 v.s. 0.96) and RMSD (30.9Å v.s. 2.4Å).

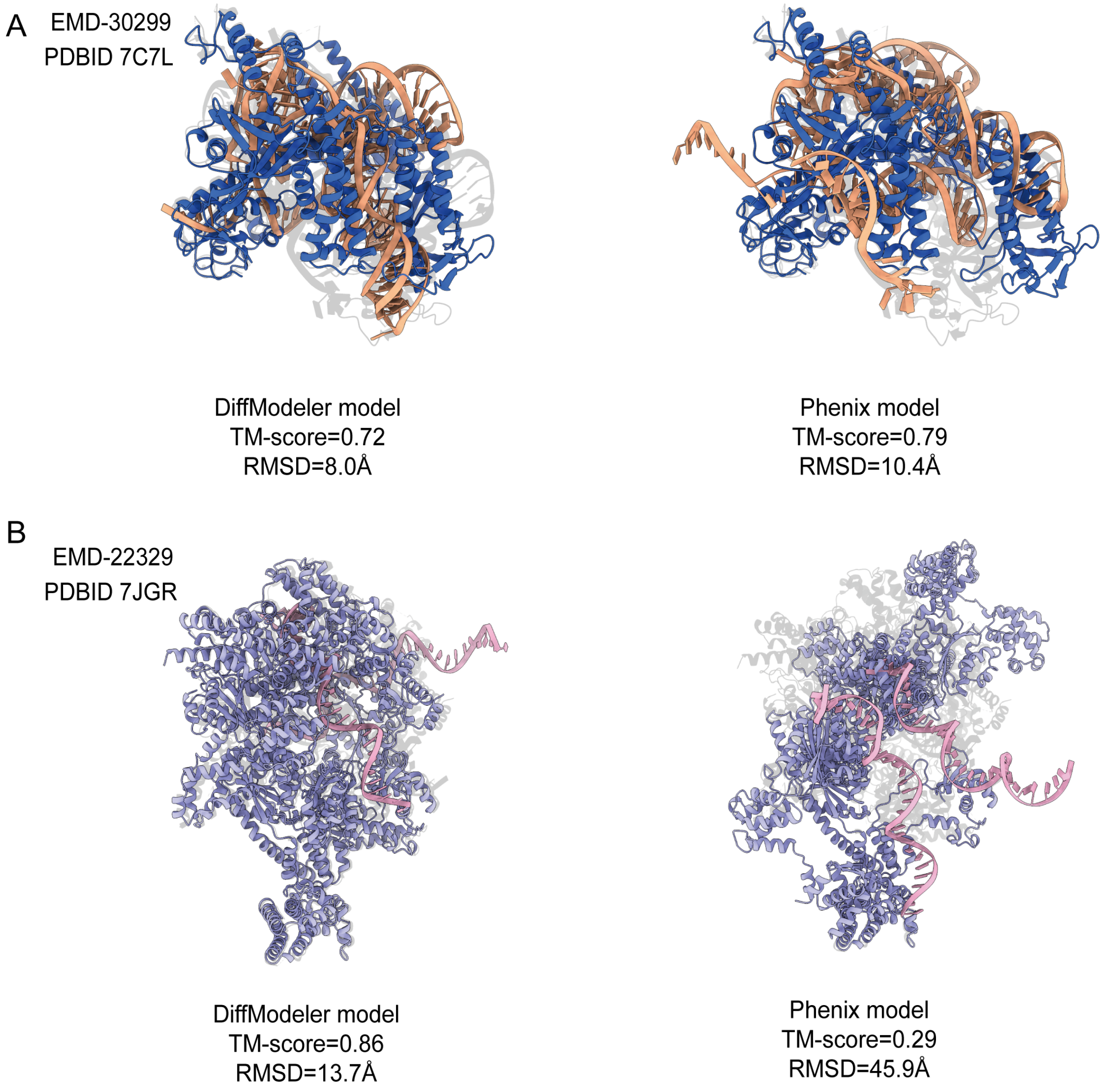

Supplementary Figure S10. Constructed models of the representative examples. **(A)** Comparison of models (blue and yellow-brown) constructed by DiffModeler and Phenix from a 3.3Å density map (EMD-30299, PDBID:7C7L) with the deposited structure (gray). **(B)** Comparison of models constructed by DiffModeler and Phenix from a 3.9Å resolution density map (EMD-22329, PDB ID: 7JGR). Proteins are depicted in purple, nucleic acids in pink, and the deposited structure is shown in gray.

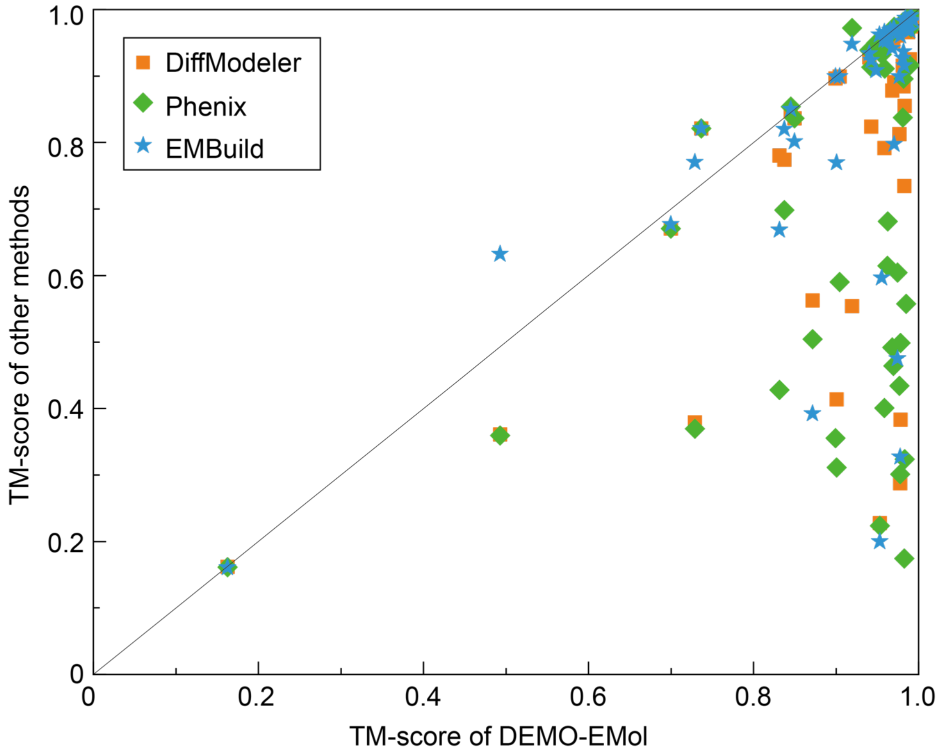

Supplementary Figure S11. Head-to-head TM-score comparison between DEMO-EMol and other methods on the 49 protein-protein complexes.

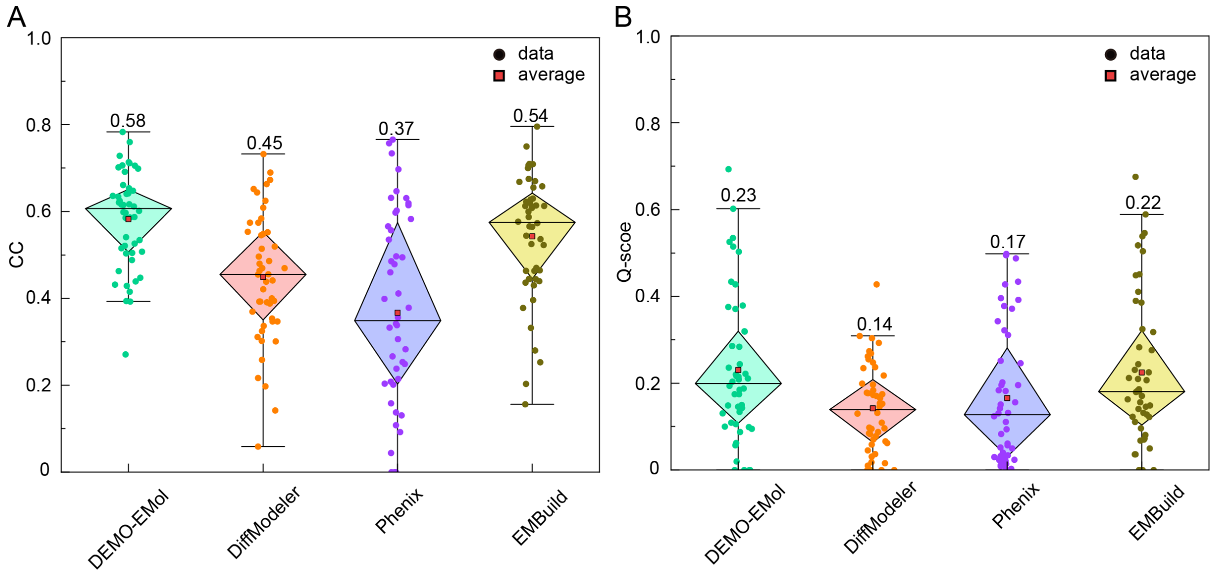

Supplementary Figure S12. Box-and-whisker plots comparing model-to-map fit metrics (CC and Q-score) across 48 protein-protein acid complexes. The diamond-shaped box represents the range from the lower to upper quartiles, with the horizontal line indicating the median and the red square denoting the means. Whiskers extend to the 5th and 95th percentiles. **(A)** CC. **(B)** Q-score.

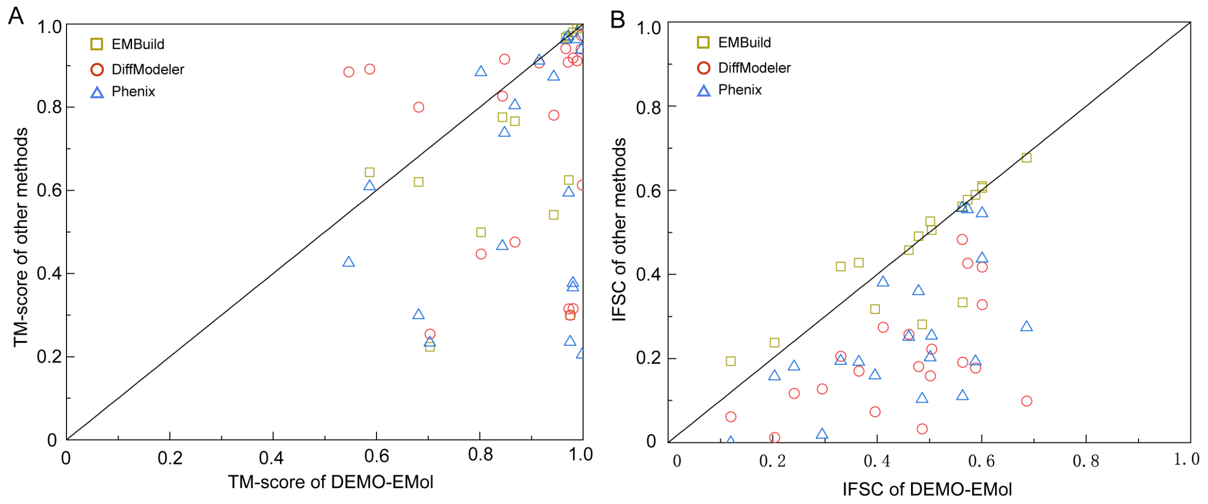

Supplementary Figure S13. Scatter plots comparing the performance of different methods on 20 density maps with resolutions <5 Å. **(A)** TM-score. **(B)** IFSC.

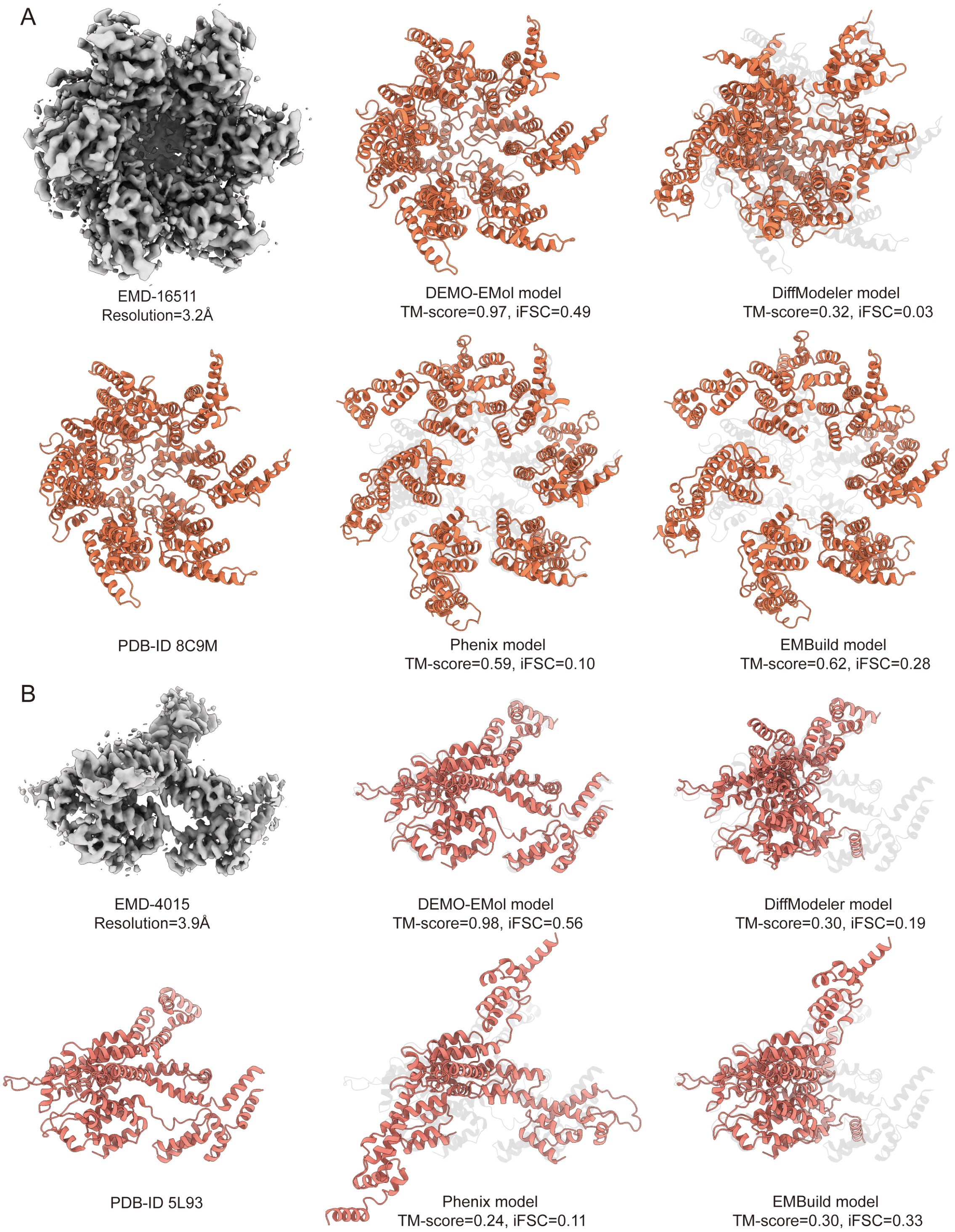

Supplementary Figure S14. Two representative examples. **(A)** Comparison of models generated by different methods (colored cartoons) with the deposited structure (semi-transparent gray) of the HERV-K Gag immature lattice (PDB ID: 8C9M, six chains). **(B)** Comparison of models constructed by different methods (colored cartoons) with the deposited structure (semi-transparent gray) for the HIV-1 CA-SP1 assembly in the presence of Bevirimat (PDB ID: 5L93 with 3 chains).

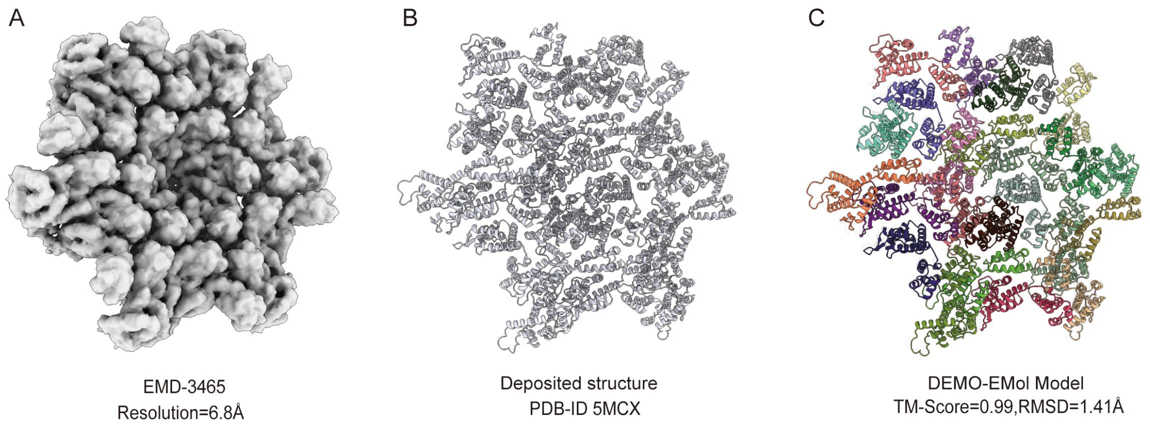

Supplementary Figure S15. The structure of the mature HIV-1 CA hexamer in intact virus particles (EMD-3456, PDBID 5MCX). **(A)** The density map. **(B)** The deposited structure. **(C)** Structural comparison between the DEMO-EMol model (colored) and the deposited structure (gray).

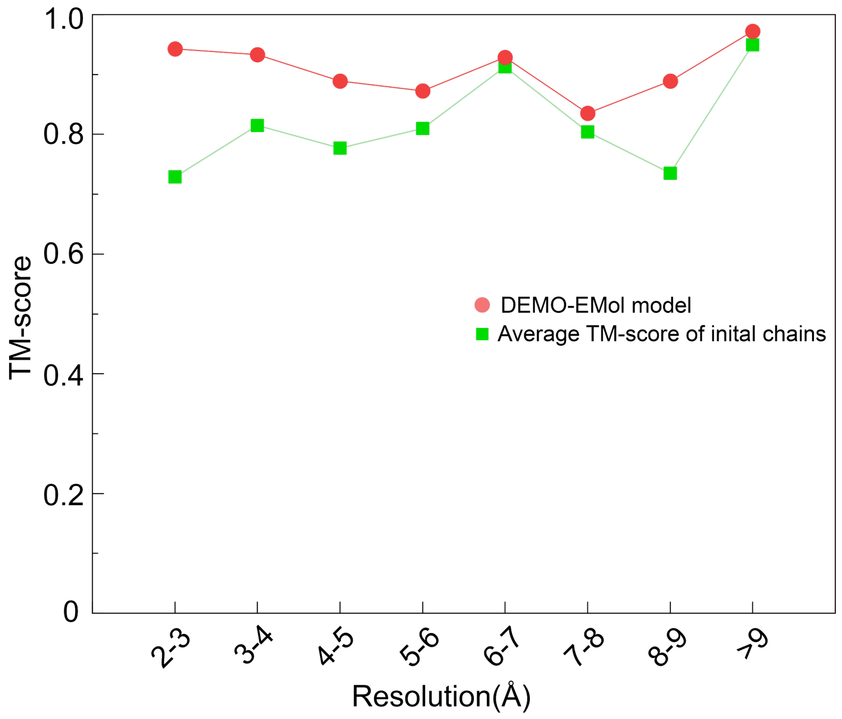

Supplementary Figure S16. The point-line plot illustrates the TM-score variation of the DEMO-EMol model across different map resolutions and the average TM-score of the initial chain structures for the 97 test cases. Red circles represent the average TM-score of DEMO-EMol models at the corresponding resolution, while green squares represent the average TM-score of the initial chain structures at the corresponding resolution.

Supplementary Tables

Supplementary Table S1. 49 protein-nucleic acid complexes.

PDBID	EMDBID	Resolution(Å)	Number of chains	Number of NA chains
5u0a	8478	3.3	14	3
5xwy	6777	3.2	2	1
5zam	6906	5.7	3	1
6ahr	9626	3.92	12	1
6b19	7031	4.5	4	2
6b44	7048	2.9	12	3
6e9f	9014	3.3	3	1
6gtf	0064	3.63	4	3
6h25	0128	3.8	12	1
6iv6	9742	3.6	2	1
6k0b	9900	4.3	14	4
6klb	0705	4.1	6	2
6lxd	30005	3.9	4	1
6mcc	9067	3.9	3	1
6mur	9253	3.1	8	2
6nm9	9398	3.38	6	2
6nud	0516	3.5	12	2
6t2c	10368	3.52	4	1
6t7c	10393	4	12	2
6uro	20861	3.6	6	1
6v4x	21050	3.2	12	2
6vpc	21308	3.2	6	3
6vrb	21366	3.0	3	1
6w6v	21564	3.0	10	1
6xmf	22257	3.1	2	1
6yov	10864	3.42	12	2
7bgb	12177	3.4	9	1
7c7l	30299	3.3	5	3
7da7	30624	3.47	3	1
7dmq	30767	3.06	3	2
7jgr	22329	3.9	9	2
7jhy	22340	3.9	12	1
7kha	22876	3.13	12	1
7lma	23437	3.3	8	2
7lys	23600	3.05	4	3
7mr4	23956	4.5	5	2
7ogm	12884	3.7	10	1
7utn	26782	2.74	4	3
7w0d	32239	4.18	6	2
7way	32389	2.9	4	3
7yzo	14391	3.4	6	2
7zqs	14874	2.54	4	2
8bmw	16126	3.5	15	1
8d4b	27180	2.92	3	2
8dc2	27320	2.99	4	3
8igr	35438	3.1	12	2
8pjj	17711	4.24	2	1
8ptg	17870	2.9	7	1
8umf	42378	2.9	5	1

Supplementary Table S2. 48 protein-protein complexes.

PDB ID	EMDB ID	Resolution(Å)	Number of chains
5kem	8241	5.5	11
5m50	4154	5.3	5
5tqw	8436	5.6	2
5u4w	8508	9.1	12
5vm7	8711	5.7	2
6kss	0774	8.1	4
6lu9	0979	8.8	4
6pwu	20511	6.2	5
6qnt	4608	3.5	4
6rgl	4876	5.4	4
6rko	4908	2.68	4
6smx	10247	6.65	5
6u9e	20695	4.21	6
6v05	20969	4.1	6
6vfi	21173	4.54	2
6vfj	21174	5.35	2
6y97	10733	4.33	4
6ytx	10925	6.23	11
7ccs	30341	6.2	2
7d3r	30565	3.49	6
7eye	31382	5.1	1
7mt8	23977	5.8	2
7peo	13355	4.37	1
7pty	13643	4.63	2
7q3y	13797	4.34	1
7q53	13824	6.3	4
7qla	14066	3.85	2
7s1m	24805	2.41	6
7sjx	25165	8.2	2
7sl9	25195	3.5	2
8bnr	16134	10.3	8
8duo	27724	5.7	6
8e3c	27863	7.1	3
8fo1	29338	3.3	4
8fzc	29607	5.5	3
8hbn	34638	3.81	2
8kgc	37217	2.54	4
8ooh	17015	7	2
8pq2	17819	3.85	3
8pwl	17994	4.73	2
8q4l	18149	5.12	3
8sl4	40573	7	2
8sta	40758	7.3	4
8usq	42511	12.77	4
8xef	38288	4.41	5
9dwu	47265	5.14	3
9ezx	50090	2.55	2
9fch	50314	6.52	2

Supplementary Table S3. 20 protein-protein complexes with maps at resolution <5Å

PDB ID	EMDB ID	Resolution(Å)	Number of chains
9b73	44299	1.96	3
9cjb	45626	1.97	4
8ror	19402	2.39	3
8zd1	39946	2.6	5
8ywe	39630	2.69	3
8ymc	39394	2.7	4
9ex7	50032	2.91	4
8puw	17949	3.02	4
8c9m	16511	3.2	6
8qkk	18464	3.23	1
8pu6	17929	3.4	3
8bqe	16183	3.5	6
6t61	10381	3.7	18
5l93	4015	3.9	3
6gzv	0103	4	4
7ash	11894	4.2	18
3j9p	6267	4.2	4
7no2	12487	4.3	3
6hv8	0287	4.4	2
6gni	0044	4.9	3

Supplementary Table S4. Results of DEMO-EMol models for the 49 protein-nucleic acid complexes.

PDBID	EMDBID	TM-score	RMSD (Å)	iFSC	CC	Q-score
5U0A	8478	0.92182	15.24	0.533277	0.578706	0
5XWY	6777	0.96929	3.93	0.519561	0.584111	0.4125
5ZAM	6906	0.87326	10.6	0.410793	0.565648	0.1535
6AHR	9626	0.96579	3.59	0.430973	0.490056	0.2925
6B19	7031	0.90514	4.96	0.186377	0.408598	0.147
6B44	7048	0.88561	12.24	0.333293	0.475485	0.203
6E9F	9014	0.99016	1.12	0.49327	0.559126	0.4355
6GTF	0064	0.96453	2.67	0.30632	0.412446	0.2855
6H25	0128	0.98513	3.1	0.639173	0.650065	0.3455
6IV6	9742	0.9397	4.62	0.480403	0.46984	0.4265
6K0B	9900	0.76643	25.41	0.383585	0.363153	0.17
6KLB	0705	0.9308	5.84	0.207769	0.349589	0.1135
6LXD	30005	0.97032	2.29	0.509324	0.614662	0.3375
6MCC	9067	0.9863	1.56	0.454014	0.545595	0.3395
6MUR	9253	0.99257	1.32	0.498945	0.566311	0.463
6NM9	9398	0.93747	8.53	0.381619	0.491884	0.3005
6NUD	0516	0.94151	6.73	0.516787	0.580935	0.2875
6T2C	10368	0.98051	2.97	0.552038	0.612582	0.365
6T7C	10393	0.86619	18.81	0.416614	0.497071	0.244
6URO	20861	0.99052	3.98	0.524727	0.578761	0.29
6V4X	21050	0.98994	2.29	0.544614	0.647402	0.4945
6VPC	21308	0.9689	12.37	0.366424	0.438366	0.3505
6VRB	21366	0.86792	11.26	0.339365	0.472851	0.3905
6W6V	21564	0.95466	5.32	0.319075	0.465444	0.3155
6XMF	22257	0.95727	2.4	0.420215	0.492874	0.354
6YOV	10864	0.81956	23.38	0.433676	0.429556	0.2975
7BGB	12177	0.96378	10.7	0.473834	0.561187	0.2995
7C7L	30299	0.95854	4.68	0.389816	0.459125	0.364
7DA7	30624	0.93637	16.31	0.489114	0.625747	0.2445
7DMQ	30767	0.95052	2.88	0.329611	0.443677	0.318
7JGR	22329	0.98098	3.18	0.570524	0.702537	0.324
7JHY	22340	0.92801	5.65	0.477432	0.484838	0.276
7KHA	22876	0.59285	11.57	0.341074	0.403323	0.164
7LMA	23437	0.74126	23.71	0.368355	0.489738	0.2055
7LYS	23600	0.992	0.95	0.514109	0.575045	0.5065
7MR4	23956	0.97527	3.96	0.501093	0.680724	0.271
7OGM	12884	0.92117	8.39	0.519157	0.599113	0.1735
7UTN	26782	0.77147	11.11	0.204013	0.316749	0.248
7W0D	32239	0.49259	16.16	0.324806	0.501231	0.1785
7WAY	32389	0.96085	3.55	0.294889	0.45784	0.3275
7YZO	14391	0.88335	9.67	0.478109	0.568845	0.2625
7ZQS	14874	0.96489	5.77	0.412301	0.64392	0.3795
8BMW	16126	0.97832	3.77	0.487801	0.680335	0.2965
8D4B	27180	0.94836	3.76	0.319529	0.481473	0.2695
8DC2	27320	0.92171	5.28	0.392197	0.481188	0.3
8IGR	35438	0.91828	13.36	0.460957	0.674856	0.304
8PJJ	17711	0.90921	8.67	0.415792	0.59171	0.2085
8PTG	17870	0.96832	3.37	0.443273	0.644619	0.252
8UMF	42378	0.96971	4.9	0.404281	0.504144	0.3745

Supplementary Table S5. Results of DiffModeler models for the 49 protein-nucleic acid complexes.

PDBID	EMDBID	TM-score	RMSD (Å)	iFSC	CC	Q-score
5U0A	8478	0.90062	11.84	0.377833	0.490358	0
5XWY	6777	0.94896	5.26	0.009817	0.108487	-0.023
5ZAM	6906	0.78929	14.41	0.265782	0.454814	0.095
6AHR	9626	0.85344	32.74	0.248898	0.37432	0.1235
6B19	7031	0.8841	8.48	0.146657	0.344558	0.098
6B44	7048	0.55529	27.03	0.182196	0.351153	0.119
6E9F	9014	0.98274	1.47	0.106482	0.286138	0.058
6GTF	0064	0.88879	5.77	0.107357	0.266672	0.0765
6H25	0128	0.96906	9.44	0.363913	0.469799	0.183
6IV6	9742	0.92189	5.87	0.38047	0.415575	0.2235
6K0B	9900	0.5707	49.28	0.268576	0.312498	0.0915
6KLB	0705	0.91562	10.38	0.146815	0.298911	0.091
6LXD	30005	0.88696	12.4	0.371005	0.5185	0.1805
6MCC	9067	0.98345	1.71	0.367308	0.491815	0.1925
6MUR	9253	0.97456	7.64	0.304956	0.426957	0.2045
6NM9	9398	0.91095	12.61	0.191744	0.348361	0.1445
6NUD	0516	0.88064	28.17	0.281361	0.405595	0.109
6T2C	10368	0.75788	17.7	0.091526	0.25964	0.03
6T7C	10393	0.62677	12.05	0.253473	0.407058	0.165
6URO	20861	0.95639	7.84	0.394541	0.48948	0.181
6V4X	21050	0.66084	15.96	0.164138	0.336906	0.112
6VPC	21308	0.77745	14.67	0.238206	0.347591	0.18
6VRB	21366	0.86353	11.72	0.21875	0.391607	0.255
6W6V	21564	0.902	13.92	0.177479	0.3642	0.1505
6XMF	22257	0.95242	2.54	0.263771	0.368749	0.175
6YOV	10864	0.62968	9.99	0.229574	0.387802	0.217
7BGB	12177	0.91562	16.07	0.271922	0.411871	0.146
7C7L	30299	0.7241	8.03	0.183467	0.325284	0.1155
7DA7	30624	0.92536	17.37	0.300175	0.489208	0.169
7DMQ	30767	0.86298	18.6	0.141471	0.288949	0.101
7JGR	22329	0.86338	13.7	0.34276	0.549109	0.158
7JHY	22340	0.84959	15.27	0.330045	0.411688	0.171
7KHA	22876	0.73053	26.38	0.285353	0.407031	0.125
7LMA	23437	0.63239	28.75	0.125147	0.284831	0.0445
7LYS	23600	0.93294	1.73	0.314825	0.457843	0.268
7MR4	23956	0.80705	8.6	0.044114	0.184433	0.01
7OGM	12884	0.90822	9.31	0.347954	0.465435	0.15
7UTN	26782	0.70372	11.6	0.097368	0.238434	0.1075
7W0D	32239	0.37211	31.96	0.196682	0.362916	0.0315
7WAY	32389	0.90439	4.27	0.144147	0.332129	0.1315
7YZO	14391	0.84947	8.88	0.290193	0.434292	0.169
7ZQS	14874	0.95269	10.41	0.242331	0.475502	0.273
8BMW	16126	0.93525	12.28	0.329815	0.527042	0.198
8D4B	27180	0.65797	15.89	0.024662	0.140446	0.0555
8DC2	27320	0.89604	4.04	0.16222	0.30001	0.112
8IGR	35438	0.81234	18.91	0.203658	0.415111	0.1345
8PJJ	17711	0.81592	16.53	0.298599	0.49002	0.09
8PTG	17870	0.9531	4.55	0.258883	0.505116	0.1905
8UMF	42378	0.91886	4.12	0.190468	0.330925	0.2015

Supplementary Table S6. Results of Phenix models for the 49 protein-nucleic acid complexes.

PDBID	EMDBID	TM-score	RMSD (Å)	iFSC	CC	Q-score
5U0A	8478	0.86165	28.58	0	0	0.202
5XWY	6777	0.93612	8.34	0.4721	0.552197	0.232
5ZAM	6906	0.80062	17.41	0.341217	0.476606	0.0745
6AHR	9626	0.95373	4.5	0.417499	0.504671	0.288
6B19	7031	0.50024	32.54	0.018927	0.0699486	0.0285
6B44	7048	0.72906	42.42	0.228471	0.354879	0.16
6E9F	9014	0.97494	1.84	0.387229	0.49122	0.2145
6GTF	0064	0.88511	8.94	0.002996	0.0343646	-0.012
6H25	0128	0.87828	13.49	0.327294	0.435684	0.285
6IV6	9742	0.9346	4.7	0.432694	0.44168	0.2075
6K0B	9900	0.44673	64.16	0.222119	0.256376	0.091
6KLB	0705	0.53792	35.65	0.081196	0.164349	0.095
6LXD	30005	0.94842	8.98	0.473951	0.59859	0.3255
6MCC	9067	0.83857	43.72	0.004516	0	0.001
6MUR	9253	0.96692	3.28	0.183655	0.331216	0.0735
6NM9	9398	0.46334	44.46	0.007385	0	0.1445
6NUD	0516	0.92712	7.69	0.237117	0.325726	0.1245
6T2C	10368	0.9713	3.25	0.504951	0.573915	0.3685
6T7C	10393	0.35125	39.42	0.166138	0.330547	0.0985
6URO	20861	0.93687	4.76	0.28063	0.356592	0.2245
6V4X	21050	0.91109	11.44	0.155147	0.337888	0.133
6VPC	21308	0.71091	31.81	0.014248	0	0.019
6VRB	21366	0.78335	37.87	0.013684	0.06791	0.007
6W6V	21564	0.92587	5.54	0.210455	0.365009	0.249
6XMF	22257	0.83826	14.93	0.21739	0.321525	0.116
6YOV	10864	0.26725	41.76	0.156309	0.250104	0.0685
7BGB	12177	0.93207	22.15	0.428759	0.531169	0.2165
7C7L	30299	0.79005	10.4	0.250388	0.360231	0.201
7DA7	30624	0.47203	47.92	0.007415	0	0.0195
7DMQ	30767	0.8539	21.43	0.16474	0.29976	0.156
7JGR	22329	0.2902	45.9	0.123828	0.229365	0.0815
7JHY	22340	0.91071	5.51	0.306847	0.370923	0.2615
7KHA	22876	0.96283	4.57	0.416939	0.456186	0.288
7LMA	23437	0.48787	42.77	0.049635	0.0919245	0.03
7LYS	23600	0.88012	14.34	0.008261	0.0526043	-0.0115
7MR4	23956	0.51396	41.62	0.179423	0.281221	0.061
7OGM	12884	0.54283	42.18	0.468621	0.536057	0.149
7UTN	26782	0.60865	9.69	0.162881	0.279852	0.1725
7W0D	32239	0.36864	33	0.187656	0.386173	0.0355
7WAY	32389	0.8123	12.37	0.016301	0.0689407	0.0815
7YZO	14391	0.81879	11.77	0.131504	0.276836	0.1285
7ZQS	14874	0.96599	5.4	0.398488	0.633615	0.389
8BMW	16126	0.69152	30.79	0.21579	0.371781	0.085
8D4B	27180	0.63025	24.35	0.002334	0.0495901	-0.007
8DC2	27320	0.83371	5.58	0.016828	0.0850226	-0.01
8IGR	35438	0.72529	36.77	0.17169	0.379255	0.094
8PJJ	17711	0.81492	16.52	0.310593	0.5018	0.171
8PTG	17870	0.95808	4.85	0.240486	0.444337	0.1465
8UMF	42378	0.85206	9.27	0.01106	0.0459678	0.036

Supplementary Table S7. Results of DEMO-EMol models for the 48 protein-protein complexes.

PDBID	EMDBID	TM-score	RMSD (Å)	iFSC	CC	Q-score
5kem	8241	0.9585	2.96	0.44878	0.620887	0.236
5m50	4154	0.98085	1.36	0.368489	0.691587	0.175
5tqw	8436	0.84523	8.7	0.192439	0.515497	0.109
5u4w	8508	0.87156	6.61	0.356972	0.534487	0
5vm7	8711	0.90423	6.32	0.216232	0.431613	0.1
6kss	0774	0.90084	7.44	0.410586	0.392567	0.088
6lu9	0979	0.95318	3.7	0.077269	0.393786	0.062
6pwu	20511	0.97033	3.19	0.256252	0.634416	0.149
6qnt	4608	0.9812	1.41	0.594741	0.653523	0.503
6rgl	4876	0.95908	6.41	0.409957	0.587312	0
6rko	4908	0.9526	2.63	0.347294	0.463122	0.693
6smx	10247	0.72871	21.6	0.313345	0.635866	0.131
6u9e	20695	0.96249	5.17	0.616058	0.648117	0.379
6v05	20969	0.97808	2.74	0.477255	0.526524	0.32
6vfi	21173	0.97467	1.16	0.429766	0.70631	0.286
6vfj	21174	0.98518	0.79	0.406404	0.713728	0.215
6y97	10733	0.95845	1.84	0.416266	0.586223	0.244
6ytx	10925	0.99387	1.28	0.702833	0.759884	0.185
7ccs	30341	0.94851	2.53	0.547989	0.611946	0.188
7d3r	30565	0.69973	8.83	0.519208	0.521229	0
7eye	31382	0.84949	6.01	0.143639	0.270959	0.057
7mt8	23977	0.16267	3.74	0.331125	0.415463	0.134
7peo	13355	0.98414	0.74	0.440226	0.705834	0.319
7pty	13643	0.8374	9.78	0.188338	0.447703	0
7q3y	13797	0.73671	29.98	0.321736	0.503771	0.22
7q53	13824	0.98888	1.08	0.645425	0.728215	0.194
7qla	14066	0.96599	2.41	0.478281	0.585994	0.371
7s1m	24805	0.98947	1.41	0.28292	0.615446	0.602
7sjx	25165	0.83149	12.11	0.702405	0.699188	0.211
7sl9	25195	0.95844	3.22	0.488743	0.596999	0.428
8bnr	16134	0.98276	2.53	0.116417	0.541105	0.106
8duo	27724	0.98326	2.34	0.445569	0.616813	0.175
8e3c	27863	0.89917	3.36	0.446206	0.439089	0.222
8fo1	29338	0.97059	3	0.543925	0.624575	0.526
8fzc	29607	0.97745	1.47	0.459302	0.640769	0.217
8hbn	34638	0.95545	2.35	0.516786	0.701518	0.376
8kgc	37217	0.98724	1.41	0.344375	0.660941	0.535
8ooh	17015	0.96789	1.59	0.379884	0.642045	0.146
8pq2	17819	0.96939	1.4	0.544356	0.616393	0.434
8pwl	17994	0.97686	2.04	0.550854	0.646161	0.209
8q4l	18149	0.91945	3.78	0.431744	0.505009	0.143
8sl4	40573	0.49275	20.28	0.069571	0.429244	0.02
8sta	40758	0.98189	2.57	0.57095	0.507701	0.095
8usq	42511	0.96228	3.66	0.237147	0.60162	0.099
8xef	38288	0.9697	1.66	0.531575	0.711312	0.284
9dwu	47265	0.94262	3.58	0.351187	0.488468	0.15
9ezx	50090	0.9908	1.61	0.283853	0.598671	0.515
9fch	50314	0.94054	3.15	0.688446	0.783353	0.204

Supplementary Table S8. Results of DiffModeler models for the 48 protein-protein complexes.

PDBID	EMDBID	TM-score	RMSD (Å)	iFSC	CC	Q-score
5kem	8241	0.94609	3.28	0.335004	0.549089	0.173
5m50	4154	0.91593	10.79	0.233548	0.552103	0.132
5tqw	8436	0.84642	8.48	0.157251	0.463734	0.098
5u4w	8508	0.56242	37.33	0.179706	0.469611	0
5vm7	8711	0.89939	6.33	0.147382	0.392651	0.083
6kss	0774	0.41342	28.47	0.131223	0.197592	0.037
6lu9	0979	0.22744	51.05	0.037291	0.142197	0.016
6pwu	20511	0.89089	14.94	0.201213	0.55334	0.13
6qnt	4608	0.96885	1.85	0.357689	0.486199	0.293
6rgl	4876	0.95634	6.37	0.351717	0.545485	0
6rko	4908	0.94198	7.16	0.22833	0.390837	0.428
6smx	10247	0.37939	30.07	0.115378	0.346639	0.062
6u9e	20695	0.95977	5.24	0.504472	0.574234	0.309
6v05	20969	0.3833	24.03	0.233861	0.388295	0.146
6vfi	21173	0.96096	1.47	0.345415	0.624868	0.237
6vfj	21174	0.96677	1.17	0.320605	0.644077	0.178
6y97	10733	0.79196	10.78	0.267576	0.441363	0.153
6ytx	10925	0.98851	1.73	0.624957	0.690098	0.166
7ccs	30341	0.93847	2.95	0.51078	0.574399	0.177
7d3r	30565	0.67083	26.87	0.227803	0.400291	0
7eye	31382	0.83618	6.15	0.081739	0.216762	0.045
7mt8	23977	0.16165	4.25	0.295283	0.353973	0.109
7peo	13355	0.98023	0.83	0.292642	0.608965	0.248
7pty	13643	0.77433	10.89	0.046949	0.311308	0
7q3y	13797	0.82099	5.87	0.088206	0.325071	0.059
7q53	13824	0.98499	1.32	0.557321	0.663542	0.171
7qla	14066	0.95654	2.63	0.349324	0.496283	0.255
7s1m	24805	0.92509	5.89	0.08726	0.346996	0.175
7sjx	25165	0.78032	13.83	0.581376	0.672768	0.154
7sl9	25195	0.95111	3.21	0.275697	0.454909	0.262
8bnr	16134	0.73452	22.92	0.023116	0.438697	0.078
8duo	27724	0.85527	11.51	0.23368	0.392591	0.085
8e3c	27863	0.89641	3.4	0.401787	0.420924	0.197
8fo1	29338	0.96375	3.23	0.312027	0.469667	0.304
8fzc	29607	0.28747	24.37	0.111738	0.301216	0.066
8hbn	34638	0.945	2.52	0.348518	0.583943	0.269
8kgc	37217	0.96584	2.18	0.115409	0.39467	0.218
8ooh	17015	0.87851	3.33	0.22963	0.514544	0.095
8pq2	17819	0.95251	1.67	0.34347	0.479804	0.274
8pwl	17994	0.81269	5.12	0.304934	0.455461	0.088
8q4l	18149	0.55396	18.49	0.257199	0.337006	0.071
8sl4	40573	0.36116	61.83	0.067907	0.0589734	0.01
8sta	40758	0.88436	7.51	0.285607	0.302483	0.018
8usq	42511	0.95103	4.22	0.209511	0.519698	0.096
8xef	38288	0.96037	1.9	0.470193	0.651921	0.235
9dwu	47265	0.82377	20.91	0.123038	0.258756	0.031
9ezx	50090	0.97477	2.43	0.113884	0.369815	0.199
9fch	50314	0.92806	3.52	0.6306	0.732231	0.184

Supplementary Table S9. Results of Phenix models for the 48 protein-protein complexes.

PDB ID	EMDB ID	TM-score	RMSD (Å)	iFSC	CC	Q-score
5kem	8241	0.40058	26.26	0.065649	0.214453	0.034
5m50	4154	0.83767	8.65	0.282801	0.535318	0.131
5tqw	8436	0.85416	8.49	0.189638	0.497044	0.111
5u4w	8508	0.50412	14.37	0	0	0.083
5vm7	8711	0.59002	12.87	0.117291	0.306441	0.057
6kss	0774	0.31142	58.17	0.045385	-0.00743844	0.009
6lu9	0979	0.22366	63.16	0.034492	0.266323	0.021
6pwu	20511	0.97378	3.09	0.264637	0.631623	0.151
6qnt	4608	0.98144	1.45	0.584309	0.646634	0.495
6rgl	4876	0.91118	7.57	0	0	0.182
6rko	4908	0.93821	3.26	0.212306	0.378438	0.392
6smx	10247	0.36968	19.02	0.029137	0.203952	0.03
6u9e	20695	0.68131	23.54	0.481949	0.478858	0.378
6v05	20969	0.49841	37.07	0.226171	0.283031	0.156
6vfi	21173	0.60438	23.13	0.042473	0.158479	0.032
6vfj	21174	0.55744	16.75	0.039423	0.249134	0.024
6y97	10733	0.95864	1.84	0.41691	0.58269	0.246
6ytx	10925	0.99382	1.29	0.697782	0.75733	0.184
7ccs	30341	0.9494	2.57	0.55854	0.61932	0.196
7d3r	30565	0.67071	19.63	0	0	0.322
7eye	31382	0.83618	6.15	0.086697	0.208139	0.052
7mt8	23977	0.16152	4.14	0.298807	0.399189	0.124
7peo	13355	0.98023	0.83	0.422902	0.696984	0.311
7pty	13643	0.69836	12.56	0	0	0.012
7q3y	13797	0.82097	5.87	-0.00265	0.0922727	0
7q53	13824	0.9902	0.99	0.649216	0.734224	0.196
7qla	14066	0.96277	2.49	0.446946	0.565832	0.343
7s1m	24805	0.91623	7.77	0.205768	0.485526	0.396
7sjx	25165	0.4276	12.39	0.103793	0.238101	0.014
7sl9	25195	0.95792	3.23	0.490551	0.59758	0.428
8bnr	16134	0.17418	45.69	-0.09935	0.341735	0.031
8duo	27724	0.32346	43.18	0.051693	0.137505	0.021
8e3c	27863	0.35567	31.5	-0.01026	0.130461	0.003
8fo1	29338	0.96908	3.03	0.513368	0.602362	0.498
8fzc	29607	0.30123	32.38	0.075505	0.201521	0.04
8hbn	34638	0.93275	2.7	0.327706	0.556903	0.252
8kgc	37217	0.97706	1.74	0.321945	0.63082	0.488
8ooh	17015	0.49192	23.19	0.004534	0.253408	0.05
8pq2	17819	0.96964	1.39	0.541801	0.614463	0.434
8pwl	17994	0.43394	45.42	0.023132	0.10811	-0.002
8q4l	18149	0.97215	2.76	0.360085	0.411323	0.132
8sl4	40573	0.3596	61.82	0.003828	0.0442124	0.011
8sta	40758	0.89596	6.97	0.320636	0.333003	0.023
8usq	42511	0.61462	33.2	0.097007	0.355869	0.061
8xef	38288	0.46379	17.41	0.183815	0.338549	0.094
9dwu	47265	0.91329	5.6	0.33553	0.460132	0.142
9ezx	50090	0.97287	2.62	0.225498	0.494349	0.372
9fch	50314	0.93881	3.21	0.676201	0.765662	0.202

Supplementary Table S10. Results of EMBuild models for the 48 protein-protein complexes.

PDB ID	EMDB ID	TM-score	RMSD (Å)	iFSC	CC	Q-score
5kem	8241	0.95431	3.13	0.433399	0.612628	0.231
5m50	4154	0.92716	1.27	0.378182	0.709568	0.186
5tqw	8436	0.84997	8.67	0.254296	0.573421	0.128
5u4w	8508	0.39243	8.86	0.284578	0.396038	0
5vm7	8711	0.89925	5.4	0.240913	0.46479	0.122
6kss	0774	0.76993	11.66	0.325732	0.280014	0.073
6lu9	0979	0.19993	57.37	0.086572	0.203096	0.036
6pwu	20511	0.79713	3.42	0.228493	0.536268	0.149
6qnt	4608	0.98249	1.41	0.594718	0.655102	0.504
6rgl	4876	0.9617	6.39	0.443931	0.612756	0
6rko	4908	0.96219	1.09	0.336316	0.463393	0.676
6smx	10247	0.77046	12.06	0.204857	0.444365	0.081
6u9e	20695	0.96358	5.18	0.634907	0.675194	0.411
6v05	20969	0.96018	3.02	0.472691	0.523095	0.318
6vfi	21173	0.47484	5.48	0.375543	0.65809	0.276
6vfj	21174	0.97035	1.1	0.379922	0.706439	0.21
6y97	10733	0.95514	1.92	0.412722	0.587229	0.244
6ytx	10925	0.98345	2.07	0.694959	0.750051	0.18
7ccs	30341	0.90848	4.19	0.521701	0.566115	0.181
7d3r	30565	0.67688	18.02	0.520627	0.564828	0
7eye	31382	0.80147	6.62	0.097667	0.253063	0.05
7mt8	23977	0.16062	4.51	0.324989	0.444864	0.141
7peo	13355	0.98689	0.67	0.43638	0.708909	0.325
7pty	13643	0.81977	10.41	0.177779	0.444909	0
7q3y	13797	0.82099	5.87	0.099865	0.332402	0.068
7q53	13824	0.97969	1.67	0.595519	0.70006	0.183
7qla	14066	0.96906	2.33	0.506372	0.608437	0.386
7s1m	24805	0.96836	4.16	0.27974	0.613916	0.589
7sjx	25165	0.66864	14.74	0.593436	0.576962	0.163
7sl9	25195	0.96631	3.1	0.512396	0.620473	0.451
8bnr	16134	0.91578	3.26	0.051959	0.436237	0.111
8duo	27724	0.97281	3.01	0.432402	0.611259	0.171
8e3c	27863	0.8992	3.4	0.440193	0.439917	0.225
8fo1	29338	0.97129	2.98	0.554719	0.629995	0.539
8fzc	29607	0.32684	19.7	0.228671	0.377901	0.123
8hbn	34638	0.59663	1.87	0.410451	0.544699	0.39
8kgc	37217	0.98465	1.21	0.352801	0.670111	0.546
8ooh	17015	0.94169	2.16	0.380816	0.627789	0.156
8pq2	17819	0.9708	1.36	0.555408	0.622408	0.449
8pwl	17994	0.89922	2.23	0.556928	0.668613	0.212
8q4l	18149	0.94799	3.23	0.350499	0.429884	0.132
8sl4	40573	0.63218	20.24	-0.00171	0.156194	0.036
8sta	40758	0.9364	5.45	0.507739	0.463649	0.07
8usq	42511	0.95361	4.06	0.279978	0.525219	0.096
8xef	38288	0.96536	1.76	0.530328	0.706698	0.283
9dwu	47265	0.92361	6.43	0.34759	0.470102	0.146
9ezx	50090	0.99099	1.6	0.284177	0.600125	0.518
9fch	50314	0.93384	3.33	0.704719	0.795926	0.207

Supplementary Table S11. TM-score of different methods for the 20 maps with resolutions <5Å.

PDB ID	EMDB ID	DEMO-EMol	EMBuild	DiffModeler	Phenix
9b73	44299	0.68136	0.62008	0.80044	0.29929
9cjb	45626	0.80239	0.49893	0.44714	0.88423
8ror	19402	0.84802	NA	0.91562	0.73825
8zd1	39946	0.86789	0.76633	0.47593	0.80402
8ywe	39630	0.58678	0.6433	0.89178	0.60924
8ymc	39394	0.84374	0.77606	0.82668	0.46558
9ex7	50032	0.54636	NA	0.88534	0.42532
8puw	17949	0.96617	0.96703	0.94093	0.9623
8c9m	16511	0.97208	0.62496	0.31547	0.59448
8qkk	18464	0.9709	0.97135	0.90848	0.97003
8pu6	17929	0.98061	0.98005	0.31594	0.37701
8bqe	16183	0.99832	0.99808	0.61264	0.20488
6t61	10381	0.99657	0.99656	0.97273	0.93773
5l93	4015	0.97511	0.2996	0.30003	0.23544
6gzv	0103	0.98828	0.98888	0.91186	0.96337
7ash	11894	0.7036	0.22361	0.25473	0.23384
3j9p	6267	0.94289	0.5413	0.78085	0.87315
7no2	12487	0.98033	0.97995	0.91851	0.36602
6hv8	0287	0.91481	NA	0.9068	0.91165
6gni	0044	0.99591	0.99518	0.94009	0.99591

Supplementary Table S12. iFSC of different methods for the 20 maps with resolutions <5Å.

PDB ID	EMDB ID	DEMO-EMol	EMBuild	DiffModeler	Phenix
9b73	44299	0.119906	0.193453	0.061775	0
9cjb	45626	0.203502	0.237614	0.0122	0.157169
8ror	19402	0.240876	NA	0.11701	0.180313
8zd1	39946	0.479398	0.490877	0.181079	0.360033
8ywe	39630	0.330355	0.418855	0.205332	0.194159
8ymc	39394	0.364755	0.427618	0.170176	0.191594
9ex7	50032	0.294817	NA	0.127905	0.018824
8puw	17949	0.601102	0.609915	0.328019	0.54481
8c9m	16511	0.485963	0.281524	0.0331	0.10329
8qkk	18464	0.573194	0.577461	0.426916	0.5546
8pu6	17929	0.46054	0.457857	0.257373	0.25112
8bqe	16183	0.686285	0.677461	0.098809	0.27395
6t61	10381	0.588074	0.589022	0.177818	0.19318
5l93	4015	0.563457	0.333223	0.191199	0.11027
6gzv	0103	0.601022	0.605503	0.417613	0.43722
7ash	11894	0.395803	0.317278	0.073504	0.15962
3j9p	6267	0.501685	0.526488	0.159262	0.20281
7no2	12487	0.504299	0.505418	0.222503	0.25403
6hv8	0287	0.411448	NA	0.2747	0.38023
6gni	0044	0.562629	0.561307	0.483311	0.5573

References

1. DiMaio, F., Tyka, M.D., Baker, M.L., Chiu, W. and Baker, D. (2009) Refinement of protein structures into low-resolution density maps using rosetta. *Journal of Molecular Biology*, **392**, 181-190.

http://www.ncbi.nlm.nih.gov/pubmed/19596339

http://dx.doi.org/10.1016/j.jmb.2009.07.008

http://www.ncbi.nlm.nih.gov/pmc/articles/PMC3899897

2. DiMaio, F., Song, Y., Li, X., Brunner, M.J., Xu, C., Conticello, V., Egelman, E., Marlovits, T., Cheng, Y. and Baker, D. (2015) Atomic-accuracy models from 4.5-Å cryo-electron microscopy data with density-guided iterative local refinement. *Nature Methods*, **12**, 361-365.

http://www.ncbi.nlm.nih.gov/pubmed/25707030

http://dx.doi.org/10.1038/nmeth.3286

http://www.ncbi.nlm.nih.gov/pmc/articles/PMC4382417

3. He, J., Lin, P., Chen, J., Cao, H. and Huang, S.Y. (2022) Model building of protein complexes from intermediate-resolution cryo-EM maps with deep learning-guided automatic assembly. *Nature Communications*, **13**, 4066.

http://www.ncbi.nlm.nih.gov/pubmed/35831370

http://dx.doi.org/10.1038/s41467-022-31748-9

http://www.ncbi.nlm.nih.gov/pmc/articles/PMC9279371

4. van Kempen, M., Kim, S.S., Tumescheit, C., Mirdita, M., Lee, J., Gilchrist, C.L.M., Söding, J. and Steinegger, M. (2024) Fast and accurate protein structure search with Foldseek. *Nature Biotechnology*, **42**, 243-246.

http://www.ncbi.nlm.nih.gov/pubmed/37156916

http://dx.doi.org/10.1038/s41587-023-01773-0

http://www.ncbi.nlm.nih.gov/pmc/articles/PMC10869269

5. Hauser, M., Steinegger, M. and Söding, J. (2016) MMseqs software suite for fast and deep clustering and searching of large protein sequence sets. *Bioinformatics*, **32**, 1323-1330.

http://www.ncbi.nlm.nih.gov/pubmed/26743509

http://dx.doi.org/10.1093/bioinformatics/btw006

6. Zhang, Y. and Skolnick, J. (2004) Scoring function for automated assessment of protein structure template quality. *Proteins*, **57**, 702-710.

http://www.ncbi.nlm.nih.gov/pubmed/15476259

http://dx.doi.org/10.1002/prot.20264

7. Xu, J. and Zhang, Y. (2010) How significant is a protein structure similarity with TM-score = 0.5? *Bioinformatics*, **26**, 889-895.

http://www.ncbi.nlm.nih.gov/pubmed/20164152

http://dx.doi.org/10.1093/bioinformatics/btq066

http://www.ncbi.nlm.nih.gov/pmc/articles/PMC2913670

8. Zhou, X., Li, Y., Zhang, C., Zheng, W., Zhang, G. and Zhang, Y. (2022) Progressive assembly of multi-domain protein structures from cryo-EM density maps. *Nature Computational Science*, **2**, 265-275.

http://www.ncbi.nlm.nih.gov/pubmed/35844960

http://dx.doi.org/10.1038/s43588-022-00232-1

http://www.ncbi.nlm.nih.gov/pmc/articles/PMC9281201

9. Takeda, S.N., Nakagawa, R., Okazaki, S., Hirano, H., Kobayashi, K., Kusakizako, T., Nishizawa, T., Yamashita, K., Nishimasu, H. and Nureki, O. (2021) Structure of the miniature type V-F CRISPR-Cas effector enzyme. *Molecular cell*, **81**, 558-570.e553.

http://www.ncbi.nlm.nih.gov/pubmed/33333018

http://dx.doi.org/10.1016/j.molcel.2020.11.035

10. Li, Z., Zhang, H., Xiao, R., Han, R. and Chang, L. (2021) Cryo-EM structure of the RNA-guided ribonuclease Cas12g. *Nature chemical biology*, **17**, 387-393.

http://www.ncbi.nlm.nih.gov/pubmed/33495647

http://dx.doi.org/10.1038/s41589-020-00721-2

http://www.ncbi.nlm.nih.gov/pmc/articles/PMC8256697
